# Biomarkers for pancreatic cancer based on tissue and serum metabolomics analysis in a multicenter study

**DOI:** 10.1002/cam4.5296

**Published:** 2022-09-26

**Authors:** Rui Zhao, Shuai Ren, Changyin Li, Kai Guo, Zipeng Lu, Lei Tian, Jian He, Kai Zhang, Yingying Cao, Shijia Liu, Donghui Li, Zhongqiu Wang

**Affiliations:** ^1^ Department of Radiology, Jiangsu Province Hospital of Chinese Medicine Affiliated Hospital of Nanjing University of Chinese Medicine Nanjing China; ^2^ Department of Clinical Pharmacology, Jiangsu Province Hospital of Chinese Medicine Affiliated Hospital of Nanjing University of Chinese Medicine Nanjing China; ^3^ Pancreas Center The First Affiliated Hospital with Nanjing Medical University Nanjing China; ^4^ Department of Nuclear Medicine, Nanjing Drum Tower Hospital The Affiliated Hospital of Nanjing University Medical School Nanjing China; ^5^ Department of Pharmacy, Jiangsu Province Hospital of Chinese Medicine Affiliated Hospital of Nanjing University of Chinese Medicine Nanjing China; ^6^ Department of Gastrointestinal Medical Oncology The University of Texas MD Anderson Cancer Center Houston Texas USA

**Keywords:** biomarker, diagnosis, metabolomics, pancreatic ductal adenocarcinoma

## Abstract

**Background:**

Early detection of pancreatic ductal adenocarcinoma (PDAC) may improve the prognosis of patients. This study was to identify metabolic features of PDAC and to discover early detection biomarkers for PDAC by tissue and serum metabolomics analysis.

**Methods:**

We conducted nontargeted metabolomics analysis in tissue samples of 51 PDAC tumors, 40 noncancerous pancreatic tissues (NT), and 14 benign pancreatic neoplasms (BP) as well as serum samples from 80 patients with PDAC, 36 with BP, and 48 healthy controls (Ctr). The candidate metabolites identified from the initial analysis were further quantified using targeted analysis in serum samples of an independent cohort of 22 early stage PDAC, 27 BP, and 27 Ctr subjects. Unconditional binary logistic regression analysis was used to construct the optimal model for PDAC diagnosis.

**Results:**

Upregulated levels of fatty acids and lipids and downregulated amino acids were observed in tissue and serum samples of PDAC patients. Proline, creatine, and palmitic acid were identified as a panel of potential biomarkers to distinguish PDAC from BP and Ctr (odds ratio = 2.17, [95% confidence interval 1.34–3.53]). The three markers showed area under the receiver‐operating characteristic curves (AUCs) of 0.854 and 0.865, respectively, for the comparison of PDAC versus Ctr and PDAC versus BP. The AUCs were 0.830 and 0.852 in the validation set and were improved to 0.949 and 0.909 when serum carbohydrate antigen 19‐9 (CA19‐9) was added to the model.

**Conclusion:**

The novel metabolite biomarker panel identified in this study exhibited promising performance in distinguishing PDAC from BP or Ctr, especially in combination with CA19‐9.

## Graphical abstract

We identified the metabolic features of pancreatic ductal adenocarcinoma (PDAC) and developed a metabolite biomarker panel for early detection of PDAC based on tissue and serum metabolomics analysis. This panel exhibited promising performance in distinguishing PDAC from benign pancreatic neoplasms (BP) or healthy controls (Ctr), especially in combination with CA19‐9.

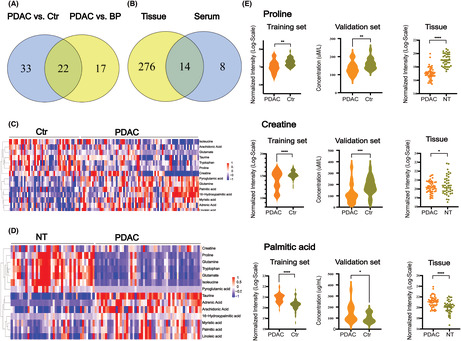

## 1 INTRODUCTION

Pancreatic ductal adenocarcinoma (PDAC) is one of the most aggressive cancers with an extremely poor prognosis, and the overall 5‐year survival rate is only 9%.^1^ However, patients with early stage PDAC who are eligible for tumor resection have a much higher survival rate.^2^ Therefore, early detection of PDAC is critical for reducing mortality and improving patient survival.

It has been reported that the time from the initial mutation in the pancreas to the development of nonmetastatic PDAC is about 10 years,^3^ which provides a wide time window for early detection. Unfortunately, accurate early diagnosis remains challenging and mainly depends on the development of highly sensitive and specific biomarkers and imaging technologies. It is widely known that carbohydrate antigen 19‐9 (CA19‐9) has insufficient sensitivity and specificity for early screening of PDAC because its level could be elevated in patients with chronic pancreatitis, cholangitis, and other gastrointestinal malignant tumors.^4^ Traditional imaging methods, such as computed tomography or magnetic resonance imaging, play important roles in preoperative diagnosis and evaluation of PDAC, but the imaging is based on anatomical and morphological changes and has limited value for early diagnosis.^5^ Therefore, more effective biomarkers with higher sensitivity and specificity for early stage PDAC are critically needed.

Metabolomics is a promising tool for discovering valuable diagnostic biomarkers and understanding carcinogenesis. Recent research have demonstrated that metabolic reprogramming is one of the hallmarks of cancers.^6^ Several studies have demonstrated the potential use of metabolites as diagnostic biomarkers for PDAC.^7^, ^8^, ^9^, ^10^ Some metabolites in biofluids, such as many amino acids (AAs), choline, and lysophosphatidylcholine have been reported to be candidate biomarkers of PDAC.^11^, ^12^, ^13^ Although great efforts have been made in the identification and development of biomarkers, there is still a lack of widely accepted reliable biomarkers for early stage PDAC.^11^, ^14^, ^15^

Currently, most metabolic research were conducted in serum, plasma, or urine samples. However, metabolite changes in biofluids are affected by several factors, including the metabolism of the liver, kidney and muscle, dietary intake, the activity of the microbiome, and other factors. Tissue metabolomics analysis is less subject to these confounding factors and can be a valuable source of metabolic biomarkers.^16^ In addition, compared with body fluids, tissue metabolism can provide metabolic deregulations more directly and can better reveal the pathogenesis of diseases and has a better chance of discovering potential diagnostic biomarkers and therapeutic targets.^17^ To our knowledge, only a few studies have used tumor tissue samples from PDAC patients for metabolomics analysis,^18^, ^19^ and their sample sizes were relatively small. Besides, comparative study on metabolic signatures of PDAC tumor tissues and biofluids is very limited.

In the current study, based on ultrahigh performance liquid chromatography equipped with quadrupole time‐off mass spectrometry (UHPLC‐Q‐TOF/MS) platform, a combined tissue and serum nontargeted metabolomics analysis was conducted using 105 pancreatic tissue samples and 164 serum samples from patients with PDAC or benign pancreatic cystic neoplasms (BP) and healthy controls (Ctr). Additionally, AAs and medium‐ and long‐chain fatty acids (FAs)‐targeted metabolomics analyses were also conducted, using serum samples of an independent cohort of 22 early stage PDAC, 27 BP, and 27 Ctr subjects, to quantify the candidate biomarkers. The primary purpose of this study is to investigate the metabolic alterations of PDAC using tissue and serum samples and to develop new potential diagnostic biomarkers.

## 2 MATERIALS AND METHODS

### 2.1 Tissue and serum sample collection

Pancreatic tissues were collected at the Pancreas Biobank of the First Affiliated Hospital with Nanjing Medical University from March to July 2019. A total of 105 pancreatic tissue samples were collected, including 51 tumors and 40 noncancerous pancreatic tissues (NT, 2 cm apart from the cancerous tissues) from PDAC patients and 14 samples from BP patients. All the tissue samples were frozen in liquid nitrogen immediately after surgical resection and stored at −80°C for further analysis. All tissue samples were pathologically confirmed.

For the training set, 164 fasting blood samples from 80 patients with PDAC, 36 with BP, and 48 Ctr were collected at three institutions (The First Affiliated Hospital with Nanjing Medical University, Affiliated Hospital of Nanjing University of Chinese Medicine, and Nanjing Drum Tower Hospital) from August 2018 to December 2019. There are 40 overlapping samples of tissue and blood from the same PDAC patients. For the validation set, 76 blood samples from an independent cohort of 22 patients with early stage PDAC, 27 with BP, and 27 with Ctr were recruited from the General Hospital of Eastern Theater Command. PDAC or BP was diagnosed pathologically after surgery, and blood samples were collected preoperatively prior to any medication. All Ctr samples were obtained from the physical examination center of Affiliated Hospital of Nanjing University of Chinese Medicine. Immediately after collection, all blood samples were centrifuged at 3000 *g* for 10 min at 4°C, and the serum samples were stored at −80°C until analysis.

This study was approved by the ethics committee of Affiliated Hospital of Nanjing University of Chinese Medicine (2017NL‐135‐05), and written informed consent was obtained from the participants. The results of laboratory tests, including the level of total cholesterol (TC), triglyceride (TG), and CA19‐9 were collected. The exclusion criteria were as follows: (1) a history of acute diseases in recent 3 months or other malignancies or recurrent PDAC, (2) concomitant autoimmune system disease or hematological disease, and (3) patients unable to give informed consent. Cancer stages were determined according to the AJCC TNM classification of malignant tumors, 8th edition.^20^

### 2.2 Sample preparation

#### 2.2.1 Pancreatic tissue sample for UPLC‐Q‐TOF/MS analysis

A piece of pancreatic tissue (100 mg) was mixed with 1 ml of 90% methanol and then homogenized by an MP homogenizer (24 × 2, 6.0 M/S, 60 s, twice). After sonicating at low temperature twice (30 min/each), the mixture was centrifuged at 4°C (13,200 *g* for 20 min). The supernatant was transferred and dried in a vacuum centrifuge and stored at −80°C. Before LC‐MS analysis, the dried supernatant was redissolved in 100 μl of acetonitrile/water (1:1, v/v), adequately vortexed, and then centrifuged at 4°C, 13,200 *g* for 15 min. Finally, the supernatant was collected for LC‐MS analysis.

#### 2.2.2 Serum sample for UPLC‐Q‐TOF/MS analysis and UPLC‐MS/MS analysis

After being thawed at 4°C, 100 μl of serum was mixed with 400 μl of cold methanol/acetonitrile (1:1, *v/v*) and vortexed for 1 min. The mixture was incubated at −20°C for 60 min and then centrifuged at 4°C (13,200 *g* for 20 min). Similar to the tissue sample preparation mentioned above, the supernatant was then transferred, dried, and redissolved for LC‐MS analysis.

#### 2.2.3 Serum sample for GC‐MS analysis

After being thawed on ice, 120 μl of serum was mixed with 1 ml of chloroform–methanol (2:1 *v/v*), and the mixture was ultrasonicated for 30 min. The supernatant was transferred and was then added to 2 ml of 1% sulfuric acid in methanol. The mixture was incubated in an 80°C water bath for 30 min to achieve fatty acid esterification. After that, 1 ml *n*‐hexane and 5 ml of water were added and mixed to extract the target compounds. Finally, 500 μl of the supernatant, spiked with the internal standard (i.e., 25 μl of 500 ppm methyl salicylate), were mixed for GC‐MS analysis.

### 2.3 Metabolomics analysis

For a nontargeted metabolomics study, the pancreatic tissue samples were analyzed using an Agilent 1290 Infinity LC system coupled to an Agilent 6550 TOF mass spectrometer and an AB Sciex TripleTOF 6600 mass spectrometer. While the serum analysis was performed on an Agilent 1290 Infinity LC system coupled to an AB Sciex TripleTOF 5600 mass spectrometer.

Serum samples were then subjected to two types of targeted metabolomics analysis to validate the potential biomarkers screened by nontargeted analysis. Of them, AAs in serum were quantified by the same Agilent 1290 Infinity LC system coupled to an AB SCIEX 5500 QTRAP mass spectrometer, while medium‐ and long‐chain FAs‐targeted metabolomics analysis was performed using an Agilent Model 7890A/5975C GC‐MS system.

Detailed information on metabolomics analysis, including methods of LC separation and MS detection for nontargeted and targeted sample analysis, is described in Supporting Information.

To monitor the stability of the analysis and ensure the precision of the results, quality control (QC) samples were prepared by mixing equal amounts of each sample in serum and tissue nontargeted and targeted metabolomics analysis, respectively.

### 2.4 Data analysis

After log transformation and pareto scaling, multivariate analysis was performed using SIMCA‐P 14.1 (Umetrics). Unsupervised principal component analysis (PCA) was performed to assess the overall metabolic alterations among groups and evaluate the stability of the analytical system. Supervised orthogonal partial least squares discriminant analysis (OPLS‐DA) was performed to compare the metabolic profiles of PDAC versus NT, BP, or Ctr. A 200‐times permutation test was subsequently used to evaluate the robustness of the model and assess the risk of overfitting for the model. The variable importance in the projection (VIP) value of each metabolite in the OPLS‐DA model was calculated to evaluate its contribution to the classification. The Student's *t*‐test or Mann–Whitney *U* test was further applied for group comparisons of metabolites with VIP >1.0. A *p* value of <0.05 was considered statistically significant. False discovery rates (FDR) were calculated using the Benjamini and Hochberg method.^9^ The metabolites that satisfied the criteria of VIP value >1.0 and FDR <0.05 were considered differential metabolites.

For targeted metabolomics analysis, the Multiquant 1.2 software (AB SCIEX) was used to extract chromatographic peak area and retention time. The standard correct retention time was used to identify the metabolites. The QC samples were processed together with the serum samples. Detected metabolites in pooled samples with a coefficient of variation of <30% were considered reproducible measurements. The metabolites that *p* < 0.05 in each comparison (PDAC vs. Ctr, PDAC vs. BP) were considered differential metabolites.

Analysis of variance (ANOVA) was performed to evaluate whether the level of metabolites was affected by potential confounding factors such as “gender,” “age,” “history of type 2 diabetes,” and “history of hypertension.” Spearman correlation analysis of these metabolites and clinical parameters, including TC, TG, and CA19‐9, was also performed. Based on the potential biomarkers, unconditional binary logistic regression was conducted to build the optimal model capable of discriminating PDAC patients from non‐PDAC participants. Subsequently, we further used unconditional binary logistic regression analysis to detect whether the model can discriminate PDAC patients from Ctr independently of clinical confounding risk factors. Finally, receiver‐operating characteristic (ROC) curve analysis was carried out to evaluate the diagnostic performance of the model. All statistical analyses were performed using R software (version 3.5.1) or SPSS 24.0 (SPSS Inc.).

Hierarchical clustering analysis (HCA) was performed using Cluster 3.0 and the Java Treeview software, and the heatmap was obtained. A correlation network was constructed using the Cytoscape software (version 3.7.1). Pathway analysis was conducted using MetaboAnalyst 4.0 and the online Kyoto Encyclopedia of Genes and Genomes (KEGG) database.

## 3 RESULTS

### 3.1 Study participants

**FIGURE 1:**
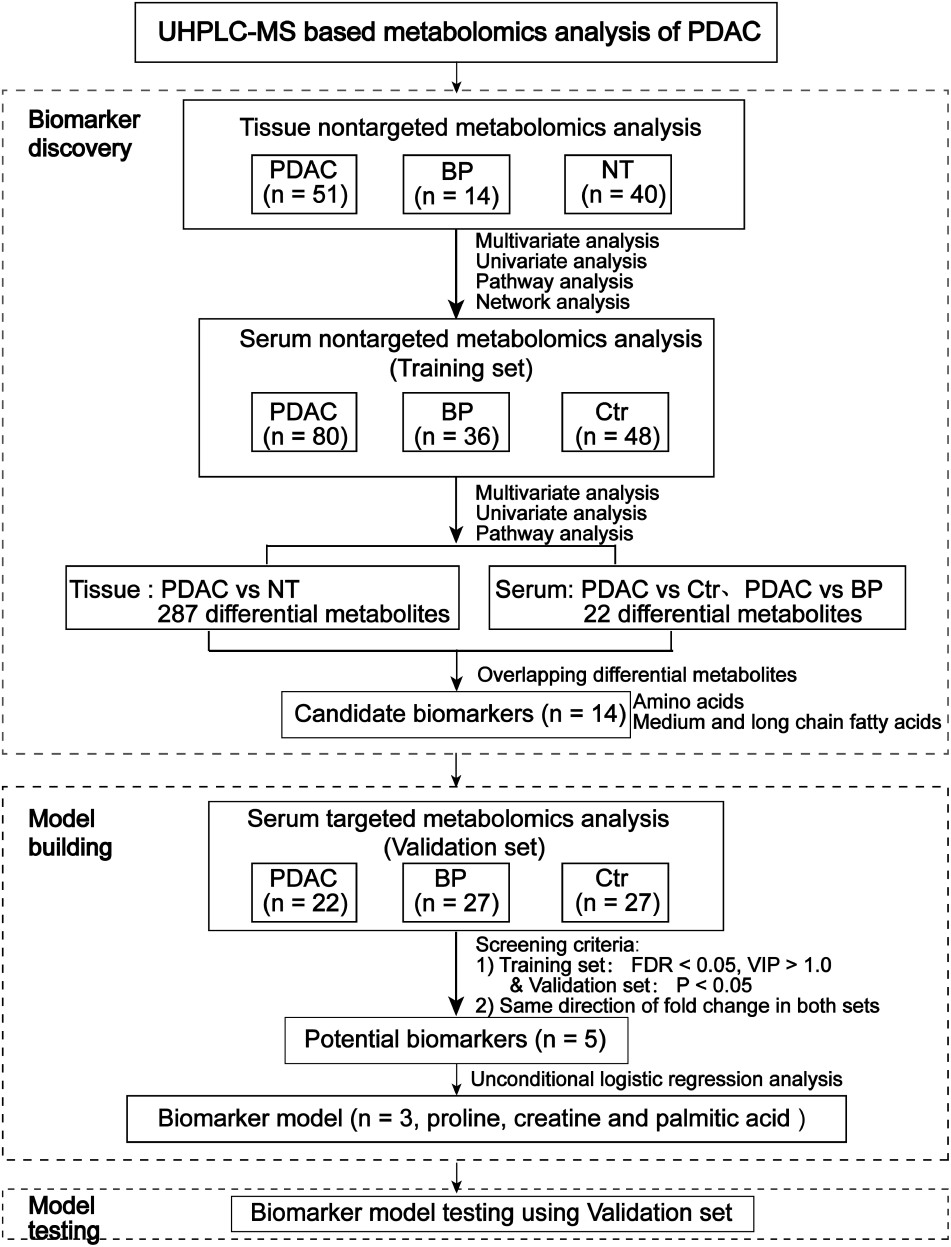
Workflow of this study. BP, benign pancreatic neoplasms; Ctr, controls; FDR, false discovery rate; NT, noncancerous pancreatic tissues; PDAC, pancreatic ductal adenocarcinoma; UHPLC‐Q‐TOF/MS, ultrahigh performance liquid chromatography equipped with quadrupole time‐off mass spectrometry

The workflow of this study is presented in Figure 1. To explore the metabolic alterations of PDAC and to identify the potential biomarkers, 105 tissue samples and 240 serum samples were analyzed. Tissue samples were obtained from 51 PDAC patients with 32 early stage (stages I and II) and 19 late‐stage diseases (stages III and IV). Paired normal adjacent tissues were available from 40 PDAC patients. The 14 patients with BP included three intraductal papillary mucinous neoplasms (IPMN), four mucinous cystic neoplasms of the pancreas (MCN), and seven serous cystadenomas of the pancreas (SCN). The BP patients are significantly younger than PDAC (*p* = 0.01).

Serum samples of the training set were from 80 patients with PDAC, 36 with BP, and 48 with Ctr. The 80 PDAC patients included 36 early stage and 44 late‐stage tumors. The 36 patients with BP included 15 IPMN, 9 MCN, and 12 SCN cases. The age between PDAC and BP groups also showed a significant difference (*p* < 0.05). Serum CA19‐9 level was routinely tested for PDAC and BP patients as well as controls. Among the 80 patients with PDAC in the training set, 20 (25%) were CA19‐9 negative (CA19‐9 < 37.0 IU/mL). Of the 36 BP patients, four were CA19‐9 positive. All control subjects were CA19‐9 negative. The validation serum samples were from an independent cohort of 22 early stage PDAC, 27 BP, and 27 Ctr. Detailed clinical characteristics of all participants are listed in Table 1.

**TABLE 1:** Clinical characteristics of all the participants in each set

Characteristics	Tissue			Serum (training set)			Serum (validation set)		
	PDAC	BP	NT	PDAC	BP	Ctr	PDAC	BP	Ctr
Total (*n*)	51	14	40	80	36	48	22	27	27
Gender (M/F)	29/22	8/6	23/17	48/32	23/13	31/17	11/11	15/12	17/10
Age (years)	60.9 ± 6.92	55.35 ± 9.00	63.35 ± 8.75	61.2 ± 6.96	58.05 ± 9.75	60.35 ± 6.19	66.91 ± 7.73	59.15 ± 10.78	58.37 ± 4.82
Location									
Head and neck	30	3		50	17		13	12	
Body and tail	21	11		30	19		9	15	
Type 2 diabetes (%)	11 (21.6)	2 (14.3)		17 (21.3)	5 (13.9)	0 (0)	6 (27.3)	6 (22.2)	0 (0)
New onset diabetes	6 (11.8)	1 (7.1)		9 (11.3)	1 (2.8)		3 (13.6)	1 (3.7)	
Hypertention (%)	9 (17.6)	4 (28.6)		22 (27.5)	13 (36.1)	4 (8.3)	7 (31.8)	11 (40.7)	0 (0)
Weight loss (%)	12 (23.5)	0 (0)		26 (32.5)	4 (11.1)	0 (0)	6 (27.3)	5 (18.5)	0 (0)
Carbohydrate antigen 19‐9 (IU/ml)	325.22 ± 364.64	14.39 ± 14.69		408.53 ± 398.45	44.59 ± 162.54	5.54 ± 5.45	564.82 ± 801.87	64.73 ± 191.54	7.98 ± 7.45
TG (mmol/L)	1.89 ± 1.03	1.93 ± 1.30		1.73 ± 1.02	1.68 ± 0.71	1.35 ± 0.77	1.80 ± 0.69	1.68 ± 0.71	1.50 ± 0.44
TC (mmol/L)	4.88 ± 1.19	4.82 ± 1.12		4.42 ± 1.31	4.81 ± 1.07	4.94 ± 0.64	4.29 ± 1.24	4.46 ± 0.74	4.63 ± 0.60
Stage (*n*)									
IA	6			5			2		
IB	7			12			6		
IIA	2			2			2		
IIB	17			17			12		
III	12			23					
IV	7			21					
BP									
IPMN		3			15			6	
MCN		4			9			7	
SCN		7			12			14	

*Note*: Data are presented as the mean ± SD.

Abbreviations: BP, benign pancreatic neoplasms; Ctr, controls; IPMN, intraductal papillary mucinous neoplasms; MCN, mucinous cystic neoplasm of pancreas; NT, noncancerous pancreatic tissues; SCN, serous cystadenoma of pancreas; TC, total cholesterol; TG, triglyceride.

### 3.2 Nontargeted metabolomics analysis

#### 3.2.1 Nontargeted metabolomics analysis of pancreatic tissue

**FIGURE 2:**
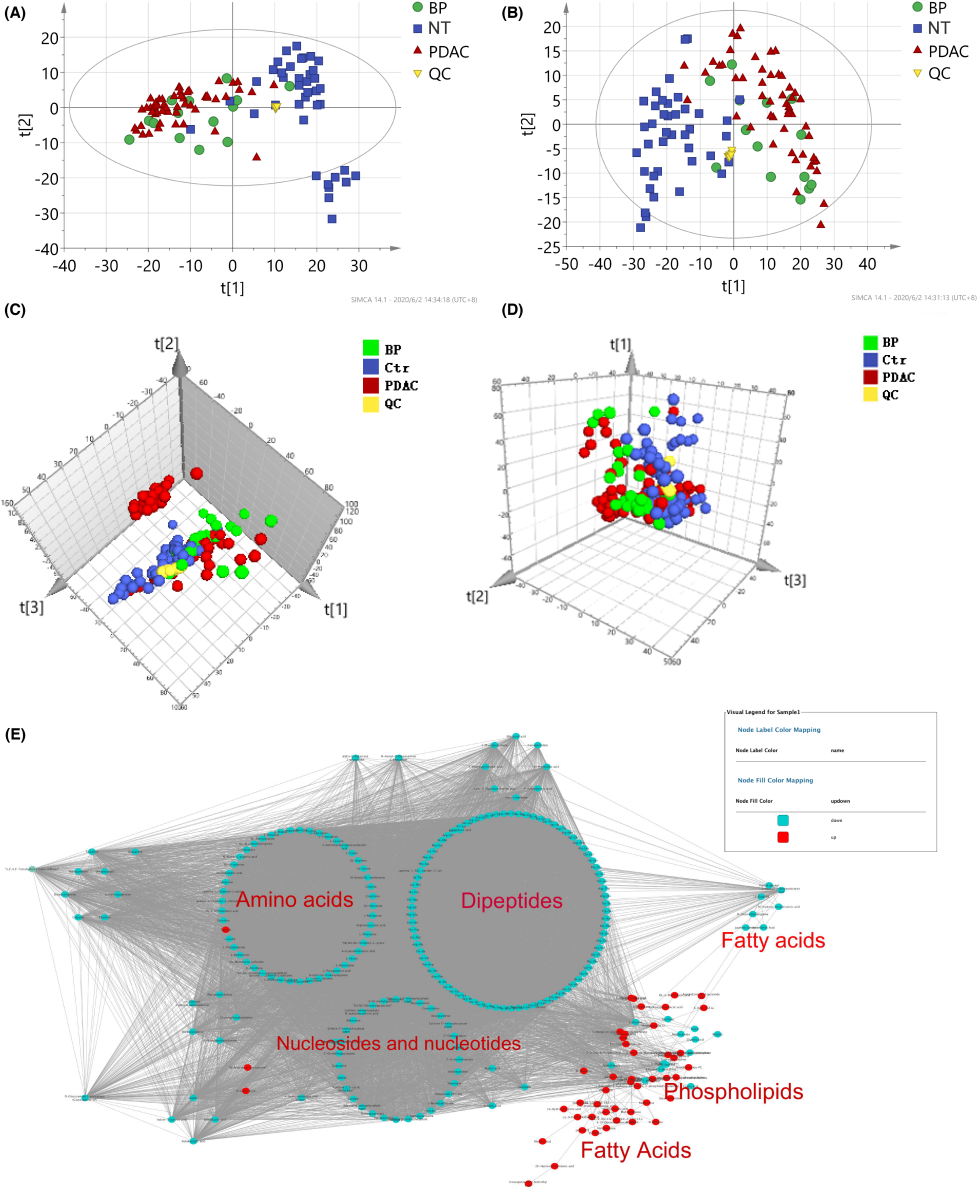
Metabolic characterization of pancreatic ductal adenocarcinoma (PDAC) using nontargeted tissue and serum metabolomics. Score plot of principal component analysis based on the data of tissue metabolomics analysis (A, positive mode and B, negative mode) and serum metabolomics analysis (C, positive mode and D, negative mode). (e) Metabolic correlation network of the differential metabolites using nontargeted tissue metabolomics. Highly correlated metabolites (|*r*| > 0.7) are connected with a line. Red node, upregulated in PDAC tumor tissue; green node, downregulated in PDAC tumor tissue. BP, benign pancreatic neoplasms; Ctr, controls; NT, noncancerous pancreatic tissues; QC, quality control

The total ion chromatograms (TIC) of QC tissue samples (Figure S1a,b) were mostly overlapped in positive and negative ion modes, respectively, indicating that the instruments were stable, and the results of metabolomics analysis were reliable. Furthermore, the PCA score plot (Figure 2A,B) showed a clear clustering of the pooled QC samples, which indicated that the sample analysis system was repeatable. In addition, the PCA showed obvious discrimination trends among PDAC, BP, and NT.

As shown in Figure S2, the OPLS‐DA model revealed clear separations of PDAC from NT, and PDAC from BP tissues without overfitting in positive and negative ion modes, respectively. Using the criteria VIP >1.0 and FDR <0.05, 287 differential metabolites were identified in PDAC tissues compared with NT (Table S3). The differential metabolites included AAs, dipeptides, nucleotides and nucleosides, FAs, lipids, glycolysis, and tricarboxylic acid cycle (TCA) metabolites. The overall heatmap of these differential metabolites is presented in Figure S3.

To obtain a global view of the metabolic alterations of PDAC, a correlation network was constructed using all the differential metabolites. It showed that extensive metabolic reprogramming had occurred in PDAC. Many FAs (stearic acid, palmitic acid, myristic acid, oleic acid, linoleic acid, arachidonic acid, etc.), lipids, glycolytic metabolites (lactate) were upregulated, whereas most AAs (l‐tryptophan, leucine, proline, arginine, aspartate) and dipeptides, nucleotides and nucleosides (uridine, xanthine, etc.), and TCA products (malate, succinate) were significantly downregulated in PDAC (Figure 2E).

Using KEGG pathway analysis, many altered metabolic pathways in PDAC were found. The most significantly perturbed pathway is central carbon metabolism in cancer, including FA biosynthesis, glycolysis, glutaminolysis, TCA, and AA metabolism (Figure S4).

#### 3.2.2 Serum nontargeted metabolomics analysis in PDAC

The TICs of the QC serum samples were also well overlapped (Figure S1c,d). Like the tissue metabolomics analysis, all the pooled QC serum samples were clustered tightly on PCA score plots in positive and negative modes (Figure 2C,D). In addition, the PCA score plots also showed significant metabolic differences among the three groups (PDAC, Ctr, and BP). OPLS‐DA score plot demonstrated the satisfactory discriminating ability of PDAC from Ctr and PDAC from BP (Figure S5a–d). Additionally, the OPLS‐DA model that distinguishes the early stage PDAC from Ctr was also developed. The score plots showed apparent separations of early 3stage PDAC from Ctr in both positive and negative modes (Figure S5e,f).

**FIGURE 3:**
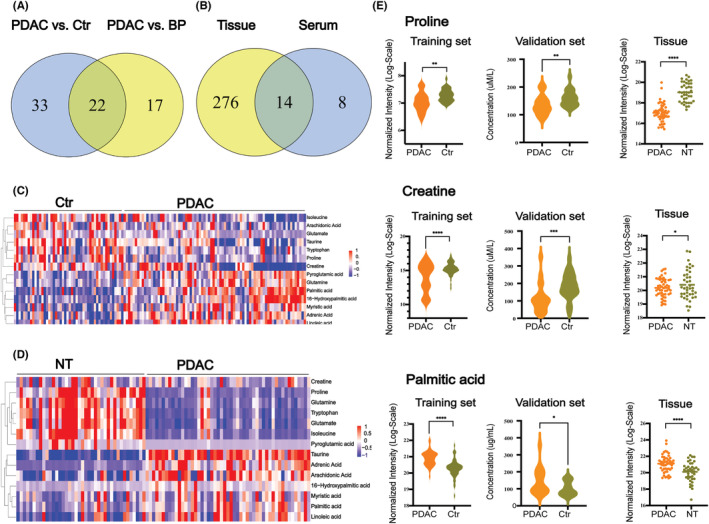
Identification of metabolite biomarkers for the diagnosis of pancreatic ductal adenocarcinoma (PDAC). (A) Venn diagram displays that 22 common differential metabolites were identified from the comparison of PDAC versus controls (Ctr) and PDAC versus benign pancreatic neoplasms (BP) in the training set in serum metabolomics. (B) Venn diagram displays 14 overlapping differential metabolites in tissue and serum samples of PDAC versus Ctr analysis. These 14 metabolites consisted of amino acids and fatty acids. (C) Heatmap of these 14 candidate metabolites between PDAC and noncancerous pancreatic tissues (NT) in tissue metabolomics analysis. (D) Heatmap of these 14 candidate metabolites between PDAC and Ctr in serum metabolomics analysis. (E) Box plot of serum relative concentrations of proline, creatine, and palmitic acid in serum and tissue metabolomics analysis. The concentration of the three metabolites shows significant differences in the comparison of PDAC and Ctr or NT (*p < 0.05, **p < 0.01, ***p < 0.001, ****p < 0.0001).

Using the criteria VIP >1.0 derived from OPLS‐DA and FDR <0.05, 55 differential metabolites in differentiating PDAC from Ctr were identified (Table S4). These differential metabolites mainly included glucose, AAs, organic acids, and lipids, 60% of which were lipids and AAs, suggesting that lipid metabolism and AA metabolism were significantly altered in PDAC. Moreover, 39 differential metabolites were identified in the PDAC group compared with the BP. Twenty‐two of the 55 (PDAC vs. Ctr) and 39 (PDAC vs. BP) differential metabolites were overlapped (Figure 3A).

#### 3.2.3 Comparative analysis of differential metabolites and perturbed pathways between tissue and serum samples

In this study, metabolomics analysis of PDAC tissue and serum showed many similar alterations in differential metabolites. Among them, 17 metabolites were altered in the same direction in PDAC tissue and serum samples. In general, the levels of various FAs (palmitic acid, myristic acid, linoleic acid, adrenic acid, arachidonic acid, etc.) and lipids were significantly increased in both tissue and serum samples of PDAC, while the levels of several AAs (tryptophan, glutamate, isoleucine, proline, creatine, taurine) and TCA products (succinate) were significantly decreased in PDAC.

Using KEGG pathway analysis based on the differential metabolites, 11 significantly perturbed metabolic pathways in PDAC versus NT or PDAC versus Ctr were commonly found in serum and tissue samples of PDAC (Table S5). The four most significantly perturbed pathways are central carbon metabolism in cancer, protein digestion and absorption, mineral absorption, and aminoacyl‐tRNA biosynthesis.

### 3.3 Identification of the potential metabolite biomarker panel

To identify potential biomarkers for validation, we first selected 22 differential metabolites in both PDAC versus Ctr and PDAC versus BP serum comparisons (Figure 3A). Then, 14 of the 22 metabolites that were significantly altered in both tissue and serum samples were initially selected to be candidate biomarkers (Figure 3B; Table S6). It was notable that the 14 metabolites included 8 AAs and 6 FAs. The heatmaps showed clearly that the 8 AAs significantly decreased and most of the FAs significantly increased in PDAC (Figure 3C,D). In the validation set, 5 of the 14 metabolites, including l‐tryptophan, proline, creatine, arachidonic acid, and palmitic acid, remained significant (*p* < 0.05) and altered in the same direction as in the training set in PDAC versus Ctr. Using ANOVA tests, the five metabolites remained significantly different between the two groups after adjusting for gender, age, history of type 2 diabetes, and hypertension (Table S7). As a result, these five metabolites were selected as potential biomarkers for further analysis.

To observe the correlation between these five metabolites and clinical characteristics, including CA19‐9, TC, and TG, Spearman correlation analysis was applied. The result showed that the levels of these five metabolites were not correlated with these clinical characteristics (Figure S6).

Subsequently, using binary logistic regression analysis, the combination of proline, creatine, and palmitic acid was developed as the biomarker panel, capable of discriminating PDAC from those non‐PDAC participants (odds ratio = 2.17, [95% CI = 1.34–3.53], *p* = 0.0017) (Table 2). To further reveal whether the panel can differentiate PDAC from Ctr independently, possible confounding factors, including gender, age, the history of type 2 diabetes, and hypertension were added to the model. It showed that the biomarker panel remained significant (*p* < 0.001) after adjustment for these clinical characteristics (Table S8). Finally, the combination of proline, creatine, and palmitic acid was defined as the potential biomarker panel to distinguish patients with PDAC from those without PDAC.

**TABLE 2:** Logistic regression analysis of pancreatic ductal adenocarcinoma‐associated serum metabolite model in the training set

Metabolite	Coefficient	Standard error	Odds ratio (95% CI)	*p* value
Intercept	0.7755	0.2474	2.1717 (1.3372–3.5268)	0.0017
Proline	−0.6988	0.2506	0.4972 (0.3042–0.8125)	0.0053
Creatine	−0.5388	0.3030	0.5834 (0.3222–1.0566)	0.0754
Palmitic acid	1.3005	0.3445	3.6711 (1.8688–7.2117)	0.0002

As shown in Figure 3E, the concentrations of proline, creatine, and palmitic acid had the same change direction in PDAC serum (training set and validation set) and tissue compared with Ctr or NT. The level of proline and creatine decreased, while palmitic acid increased.

### 3.4 Diagnostic performance evaluation of the biomarker panel

**FIGURE 4:**
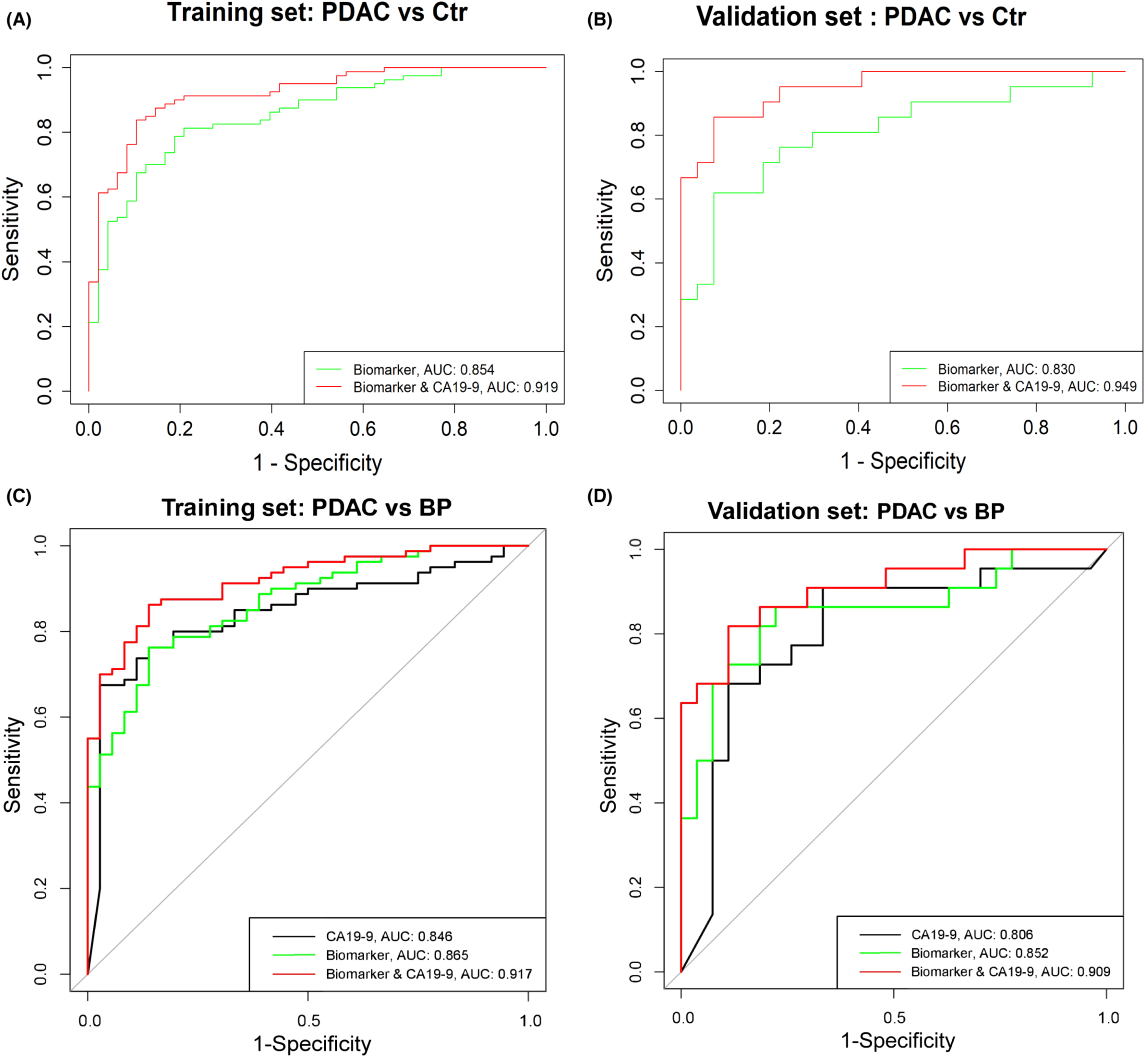
Diagnostic performances of the biomarker panel. (A, B) Receiver operating characteristic (ROC) curves of the biomarker panel and its combination with carbohydrate antigen 19‐9 (CA19‐9) in the comparison of pancreatic ductal adenocarcinoma (PDAC) and controls (Ctr) in the training set and validation set. (C, D) ROC curves of the biomarker panel and its combination with CA19‐9 in the comparison of PDAC and benign pancreatic neoplasms (BP) in the training set and validation set. AUC, area under the receiver‐operating characteristic curve

The diagnostic performance of the three metabolites panel was evaluated in the training set and validation set (Table S9). In the training set, the biomarker panel yielded an area under the receiver‐operating characteristic curve (AUC) value of 0.854 (95% CI = 0.842–0.865) in the comparison of PDAC and Ctr (Figure 4A) and 0.865 (95% CI = 0.800–0.931) in the comparison of PDAC and BP (Figure 4C). In the validation set with all early stage PDAC, the biomarker panel yielded AUCs of 0.830 (95% CI = 0.792–0.864) (Figure 4B) and 0.852 (95% CI = 0.736–0.967) (Figure 4D) in the two comparisons. This panel had a higher diagnostic performance than did CA19‐9 in the differentiation of PDAC from BP, with AUCs of 0.865 (95% CI = 0.800–0.931) and 0.852 (95% CI = 0.736–0.967) for the panel versus 0.806 (95% CI = 0.719–0.892) and 0.757 (95% CI = 0.616–0.897) for CA19‐9 in the training set and validation set, respectively. Notably, the combination of the panel with CA19‐9 can robustly distinguish PDAC from Ctr or BP, with AUCs of 0.919 and 0.949 (PDAC vs. Ctr), 0.917 and 0.909 (PDAC vs. BP) in the training set and validation set, respectively.

Additionally, we also assessed the performance of the panel in discriminating early stage PDAC patients (*n* = 36) from Ctr in the training set. The panel achieved a higher AUC of 0.880 (95% CI = 0.864–0.896), and a higher sensitivity of 88.9% (Figure S7a) than those in the analyses of PDAC patients with all tumor stages (*n* = 80). Besides, for the CA19‐9‐negative patients with PDAC (*n* = 20) in the training set, the AUC of the panel (Figure S7b) was 0.851 (95% CI = 0.840–0.863), and the accuracy was 0.72.

## 4 DISCUSSION

In the present study, we analyzed 105 tissue samples from patients with PDAC and BP as well as 240 serum samples from PDAC, BP, and Ctr using the UHPLC‐Q‐TOF‐MS platform. As far as we know, in the study of metabolomics analysis using PDAC tumor tissues, our sample size is the largest to date. Furthermore, in this study, many similar alterations of differential metabolites and perturbed metabolic pathways were found in both PDAC tissue and serum samples. FAs including palmitic acid, myristic acid, linoleic acid, adrenic acid, arachidonic acid, and lipids were upregulated, whereas most AAs (tryptophan, glutamate, isoleucine, proline, creatine, taurine) and TCA products (succinate) were significantly downregulated in PDAC. After systematic analyses of the data of PDAC tissue and serum metabolomics, a biomarker panel consisting of proline, creatine, and palmitic acid was identified and validated.

The upregulation of FAs and lipids in our study may be attributed to the increase of various lipases and de novo FA synthesis that is observed in PDAC cells and many other cancers.^21^, ^22^ These alterations are to satisfy the increasing demand for energy and building blocks for cell membrane formation during rapid cell proliferation.^23^ Free FAs play important roles in numerous biological processes. They serve as a source of energy and precursors of many signaling and inflammatory molecules. Additionally, increased levels of adrenic acid and arachidonic acid were observed in previous reports.^13^, ^24^ However, our findings on the alteration of FAs are not in accordance with previous studies.^25^, ^26^ Zhang et al. performed an integrated metabolomics and transcriptomics study using PDAC cancer tissues.^25^ Although they have elucidated that the lipid metabolism network was significantly altered in PDAC, many lipolytic enzymes and FAs, including palmitic acid and stearic acid, were decreased in tumors compared with adjacent noncancerous tissues in their study. Possible explanations of the difference could be that the PDAC samples they used may be of different metabolic subtypes from ours, that the lipolytic enzymes and free FAs had been rapidly metabolized to synthesize membrane phospholipids in their tissue samples, or that the results were affected by several confounding factors of metabolism (such as dietary or different races). These different results may indicate the remarkable complexity of metabolic alterations of PDAC.^27^ Further research are needed to elucidate the changes in lipid metabolism and metabolic subtypes of PDAC.

The downregulation of AAs indicated that the anabolism of protein was vigorous, with enhanced uptake of AAs to satisfy the rapid proliferation of cancer cells. These findings are consistent with a previous study using tissues from PDAC patients (*n* = 15),^18^ which showed that creatine and leucine were significantly downregulated in PDAC. Additionally, the alterations of AAs identified in our study are consistent with other clinical studies using serum or plasma.^7^, ^9^, ^28^ Mayerle et al. found that many AAs were significantly downregulated in PDAC, including proline, histidine, arginine, glutamine, and creatine.^9^ Fukutake et al. conducted an AAs‐targeted metabolomics study using plasm samples from 360 patients with PDAC and 8372 healthy control subjects by the HPLC‐MS platform.^28^ They found that the concentrations of 14 AAs (threonine, asparagine, proline, histidine, leucine, etc.) were significantly decreased and the concentration of serine was significantly increased in PDAC patients compared to healthy control subjects (all *p* < 0.05). Although there were some discrepancies in the concentration changes of several AAs, most AAs were downregulated in PDAC patients according to previous studies.^9^, ^10^, ^28^, ^29^ In addition, several prospective studies found that the concentrations of BCAAs,^30^, ^31^ dipeptide, aspartic acid, and glutamate^32^ were positively correlated with the risk of PDAC. These independent studies point to the potential use of AAs as biomarkers for PDAC detection.

Based on the consistency of tissue and serum metabolomics analysis in the present study, using combined nontargeted and targeted technology, a biomarker panel consisting of proline, creatine, and palmitic acid was developed. To our knowledge, several studies aimed to explore the new biomarkers of PDAC have been conducted.^7^, ^8^, ^9^, ^10^, ^11^, ^33^, ^34^, ^35^, ^36^, ^37^ However, most of them utilized biofluids, such as serum,^7^, ^33^ plasma,^8^, ^34^ urine,^35^, ^36^ or saliva^37^ samples, which can be easily affected by many extrinsic confounding factors. In the present study, we analyzed both tissue and serum samples from PDAC patients with relatively larger sample sizes. Notably, 40 overlapping samples of tissue and blood from the same PDAC patients were used in the nontargeted analysis. The combination of tissue and serum metabolomics analysis may give us a systematic understanding of PDAC metabolic reprogramming and may provide potential reliable diagnostic biomarkers.

The biomarker panel identified in our study showed promising performance in discriminating PDAC patients from Ctr or BP in the training set and validation set. In the comparison of PDAC and BP, the panel had higher diagnostic performance than CA19‐9. Furthermore, our results showed that the combination of the panel with CA19‐9 had excellent discriminative ability in both sets. Recent studies have reported several blood‐based potential biomarkers for PDAC, including AAs, bile acids, FAs, and lipids.^9^, ^11^ In this study, the level of proline was significantly decreased in patients with PDAC compared with Ctr. The result is consistent with a previous report.^9^ Reduced proline was found in their constructed biomarker panel (nine metabolites and CA19‐9) for the differential diagnosis between PDAC and chronic pancreatitis.^9^ The reason for the reduced level of proline remains unclear. Possible explanations may be the result of enhanced uptake and utilization of AAs in the cancer cell proliferation or the result of malnutrition of PDAC patients. Proline is a nonessential AA, which plays an important role in the production of extracellular matrix collagen, facilitating tumor invasion.^6^, ^38^ It has been reported that proline biosynthesis, which acts as a redox vent, can prevent the generation of destructive reactive oxygen species (ROS) and maintain the redox steady state of tumor cells.^39^ Creatine is an endogenous AA derivative and is vital for energy storage. It can be phosphorylated to creatine phosphate, which serves as a phosphate donor in the conversion of ADP to ATP. As observed in our study, a reduced level of creatine has been previously reported in plasma and urine samples of PDAC patients.^19^, ^36^ According to KEGG annotation, creatine also participates in the metabolism of glycine, serine, threonine, arginine, and proline. Palmitic acid is a saturated long‐chain FA, which is the main product of FA synthase (FASN).^40^ The upregulated palmitic acid is correlated with the elevated serum level of FASN.^40^ Di Gangi et al. found that palmitic acid showed a high AUC value in distinguishing patients with PDAC from Ctr in their study.^41^ Emerging evidence indicates that activated lipid biosynthesis is required for cancer cell growth, and FAs can be served as potential biomarkers.

According to pathway analysis, more than 10 overlapping pathways were observed in both tissue and serum samples in our study. Of which, tumor central carbon metabolism, including glycolysis, glutaminolysis, AA metabolism, FA biosynthesis, and nucleotide metabolism, was the most significantly perturbed metabolic pathway in PDAC. The result is partially consistent with other previous reports.^18^, ^42^, ^43^ According to Wang et al.,^18^ the perturbed pathway in PDAC patients is mainly related to glycerophospholipid metabolism, glycolysis, and gluconeogenesis. Several reports have revealed that enhanced glutaminolysis can provide sufficient NADPH to support FA biosynthesis and can be a source of nitrogen for the biosynthesis of nucleotides and nonessential AAs.^43^ The identification of differential metabolites and pathways of PDAC tissue and serum may give some insight into the underlying mechanisms.

This study has several limitations. First, the sample size is insufficient, especially for stage I PDAC. The current panel requires further validation in independent cohorts consisting of more stage I PDAC patients. Second, the BP group included patients with pancreatic cystic tumors (MCN and IPMN) that were at risk for PDAC, but missing other populations, such as chronic pancreatitis and new‐onset diabetes. Third, further validation including other malignant tumors, such as pancreatic neuroendocrine carcinoma, cholangiocarcinoma, ampullary cancer, and gastrointestinal malignant tumor, is needed to evaluate the specificity of this panel for PDAC. Besides, the qualitative evaluation was insufficient. Of the 14 candidate metabolite biomarkers, pyroglutamic acid and 16‐hydroxypalmitic acid were not validated in this study, which need to be qualified in the future.

In summary, tissue and serum metabolomics analysis of PDAC in our study showed similar metabolic changes and perturbed pathways. Based on them, a biomarker panel consisting of proline, creatine, and palmitic acid was developed and could serve as a promising biomarker panel or a complementary role to CA19‐9 for the detection of PDAC.

## AUTHOR CONTRIBUTIONS

Rui Zhao: project conception, methodology, formal analysis, and writing of the original draft. Shuai Ren: methodology and formal analysis. Changyin Li: methodology and data curation. Kai Guo: investigation and validation. Zipeng Lu, Lei Tian, and Jian He: Resources. Kai Zhang: data curation. Yingying Cao: Software. Shijia Liu: project conception. Donghui Li: Project conception, review, and revised the manuscript. Zhongqiu Wang: project conception and supervision.

## FUNDING INFORMATION

This work was funded by grants from the National Natural Science Foundation of China (no. 82202135, 82171925), Developing Program for High‐Level Academic Talent in Jiangsu Hospital of TCM (no. y2018rc04), and Innovative Development Foundation of Department in Jiangsu Hospital of Chinese Medicine (grant no. Y2019CX27 and Y2021CX19).

## CONFLICT OF INTEREST

The authors declare that they have no potential conflict of interest.

## ETHICS STATEMENT

This study was approved by the ethics committee of Affiliated Hospital of Nanjing University of Chinese Medicine (2017NL‐135‐05).

## PATIENT CONSENT STATEMENT

Written informed consent was obtained from all the participants.

## Supporting information


Appendix S1.

## Data Availability

Data are available upon reasonable request from the following e‐mail address: zhongqiuwang@njucm.edu.cn.
