# Supplementary material for: Biomarkers for pancreatic cancer based on tissue and serum metabolomics analysis in a multicenter study
Source: Cancer Med. 2022 Sep 26;12(4):5158–71. doi: 10.1002/cam4.5296 (PMC9972159; doi:10.1002/cam4.5296)
Supplement: Supplementary file 1 — Appendix S1. [file CAM4-12-5158-s001.docx]

Supporting information

# Supplementary methods

# Metabolomics analysis

# 1. Nontargeted tissue UPLC-Q-TOF/MS analysis

Chromatographic separation was performed using a UHPLC system (Agilent 1290 Infinity LC, Agilent Technologies, California, USA) equipped with an ACQUIY UPLC BEH HILIC column (2.1 mm × 100 mm, 1.7 µm, waters, Ireland). The column temperature was set at 25°C. The mobile phase A was 25 mM ammonium acetate and 25 mM ammonium hydroxide in water and the mobile phase B was acetonitrile. The elution gradient was started with 95% B for 0.5 min and linearly reduced to 65% at 7 min, and then to 40% at 8 min and maintained for 1 min, and then linearly increased to 95% in 0.1 min, and finally was held for 2.9 min. The flow rate was 0.3 mL/min. Volumes of 2 µL were injected using an autosampler thermostatted at 4°C.

Mass detection was conducted on two TOF mass spectrometers. First, Agilent 6550 mass spectrometer (Agilent Technologies, California, USA) was used to acquire the precursor ion information for the metabolite quantitation. Then, AB TripleTOF 6600 mass spectrometer (AB SCIEX, California, USA) was employed to obtain product ion information for the metabolite identification. The ESI source conditions of Agilent 6550 were set as follows: gas temperature: 250°C; drying gas flow: 16 L/min; nebulizer: 20 psi; sheath gas Tem: 400°C; sheath Gas Flow: 12 L/min; vcap: 3 KV; nozzle voltage: 0 V; fragment:175 V; mass range: 50-1200 Da; acquisition rate: 4 Hz; circulating time: 250 ms. While the ESI source parameters of TripleTOF 6600 were set as follows: Ion Source Gas1: 40 psi; Gas2: 80 psi; curtain gas: 30 psi; source temperature at 650°C; IonSpray Voltage Floating (ISVF): ± 5 KV. The product ion scan was acquired using information-dependent acquisition (IDA) with high sensitivity mode. Declustering potential: ± 60 V; collision voltage set at 35 ± 15 eV. IDA was set as follows: exclusion isotope within 4 Da, the maximum number of candidate ions to monitor per cycle: 10. MetDDA and LipDDA methods were used to identify the metabolites of the collected data.

# 2. Nontargeted serum UPLC-Q-TOF/MS analysis

Serum LC-MS analysis was performed using a UHPLC system (Agilent 1290 Infinity LC, Agilent Technologies, California, USA) coupled to a TripleTOF 5600 mass spectrometer (AB SCIEX, California, USA). The column type and temperature, the components and flow rate of mobile phase, the autosampler temperature, and the injection volume were the same as tissue LC-MS analysis mentioned above. The elution gradient of mobile phase started with 95% B for 1 min and linearly reduced to 65% during the following 13 min, and then linearly reduced to 40% at 16 min and held for 2 min, and then linearly increased to 95% in 0.1 min, and was held for 5 min. The ESI source parameters for mass detection were set as follows: Ion Source Gas1: 60 psi; Gas2: 60 psi; curtain gas: 30 psi; source temperature: 600°C; ISVF: 5.5 KV or - 4500 V. TOFMS scan conditions were as follows: mass range: 60-1000 Da; accumulation time: 0.20 s. The product ion scan was acquired using IDA with high sensitivity mode. Major IDA switch criteria were as follows: exclusion isotope within 4 Da, maximum number of candidate ions to monitor per cycle: 6. For Product Ion scan type, TOF mass range was set at m/z 25-1000, accumulation time was set at 0.05 s, declustering potential: ± 60 V; collision voltage set at 35 ± 15 eV, ion release delay at 67 ms, ion release width at 25 ms, and the other parameters were same with TOFMS scan type.

# 3. Targeted serum UPLC-MS/MS analysis

Free amino acid targeted analysis was conducted by Agilent 1290 Infinity LC coupled to a QTRAP 5500 (AB Sciex, California, USA). Amino acids were separated on a Zic-HILIC 3.5μm, 2.1 mm×150 mm column (waters, Ireland) with a gradient elution at a flow rate of 0.25 mL/min. The mobile phase A was 25 mM CH3COONH4+0.08% FA in water and the mobile phase B was 0.1% FA in acetonitrile. The gradient started with 90% B and linearly reduced to 70% at 12min, and then linearly reduced to 50% at 18min and reduced to 40% at 25min, and held until 30min, then the B increased to 90% in 0.1min and kept for 90% at 30.1-37min. The column oven temperature was maintained at 40°C, while 1 µL aliquot of each sample was injected using an autosampler thermostatted at 4°C. The quantification of free amino acids in the validation set was performed with reference to their internal standards.

The QTRAP 5500 (AB Sciex, California, USA) equipped with ESI source in positive ion mode was employed for MS detection. The major source/gas parameters were set as follows: source temperature 500℃, ion Source Gas 1: 40 psi, Gas 2: 40 psi, Curtain gas: 30 psi, ISVF: 5500 V. Seven free amino acids were detected by their corresponding precursor-product ion pair (as shown in Table S1_SuppInfo) in multiple-reaction monitoring (MRM) mode.

# 4. Targeted serum GC-MS analysis

GC-MS analysis was performed using an Agilent Model 7890A/5975C GC-MS system. To quantify medium- and long-chain fatty acid, Supelco FAME (fatty-acid methyl ester) mix (Sigma-Aldrich) was used to construct a calibration curve for the concentration range of 0.5–1000 mg/L. The internal standard was used to correct for injection variability between samples and minor changes in the instrument response.

An Agilent DB-WAX capillary GC column (30m × 0.25mm ID × 0.25µm) was used to separate the sample. The temperature started at 50°C and held for 3 min, then increased to 220°C at 10°C/min, and remained at 220°C for 5 min. The carrier gas was helium (1.0 mL/min). Major MS parameters were as follows: The temperatures of the injection port and transmission line were 280°C and 250°C, respectively; The electron bombardment ionization source in Selected ion Monitor (SIM) scanning mode was adopted, and electron energy was set at 70 eV. The detailed information on the five FAs was summarized in Table S2_SuppInfo.

**Supplementary figures**


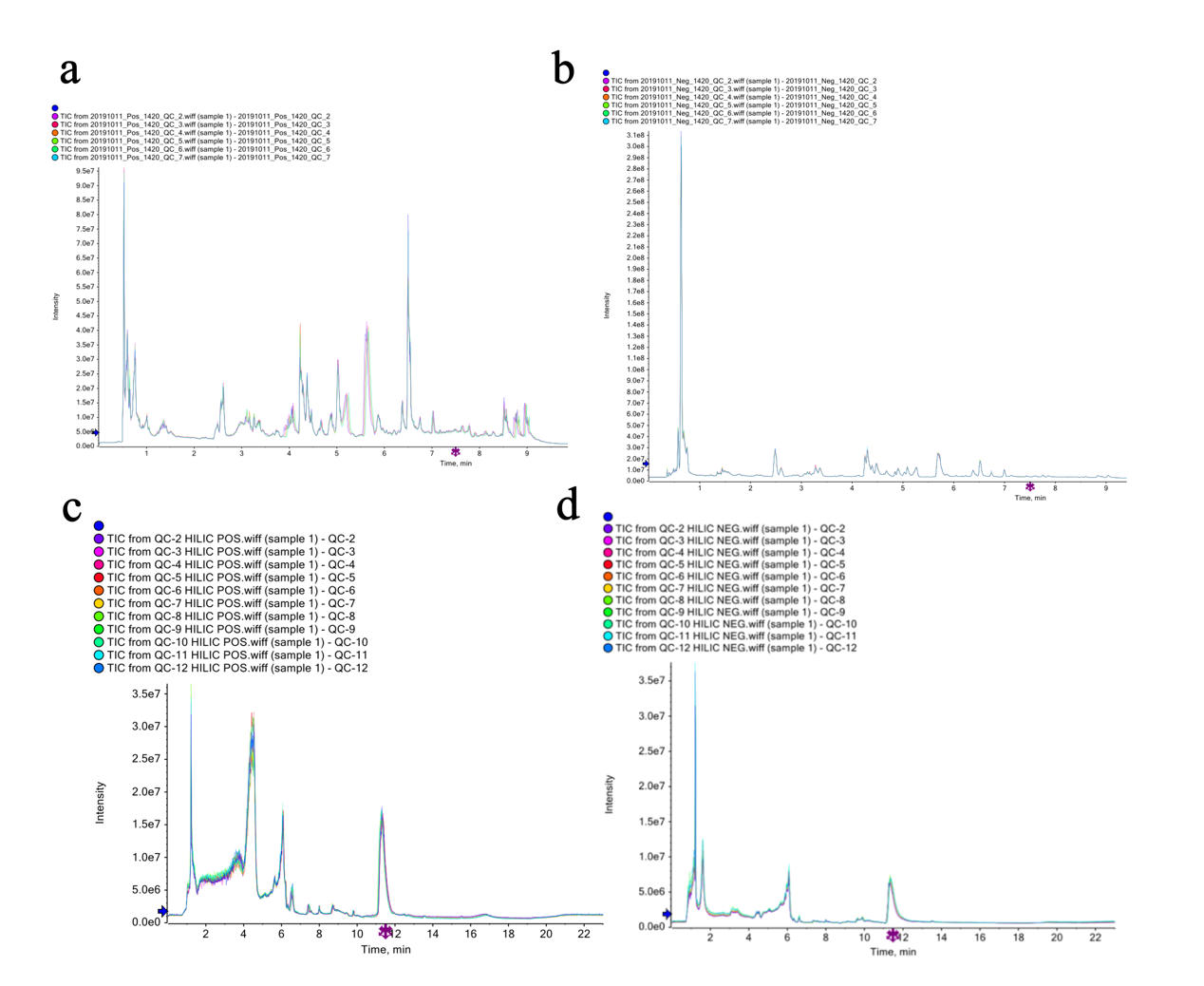


**Fig. S1 TIC of the ESI+ and ESI - modes of QC samples of tissue (A, B) and serum (C, D).**


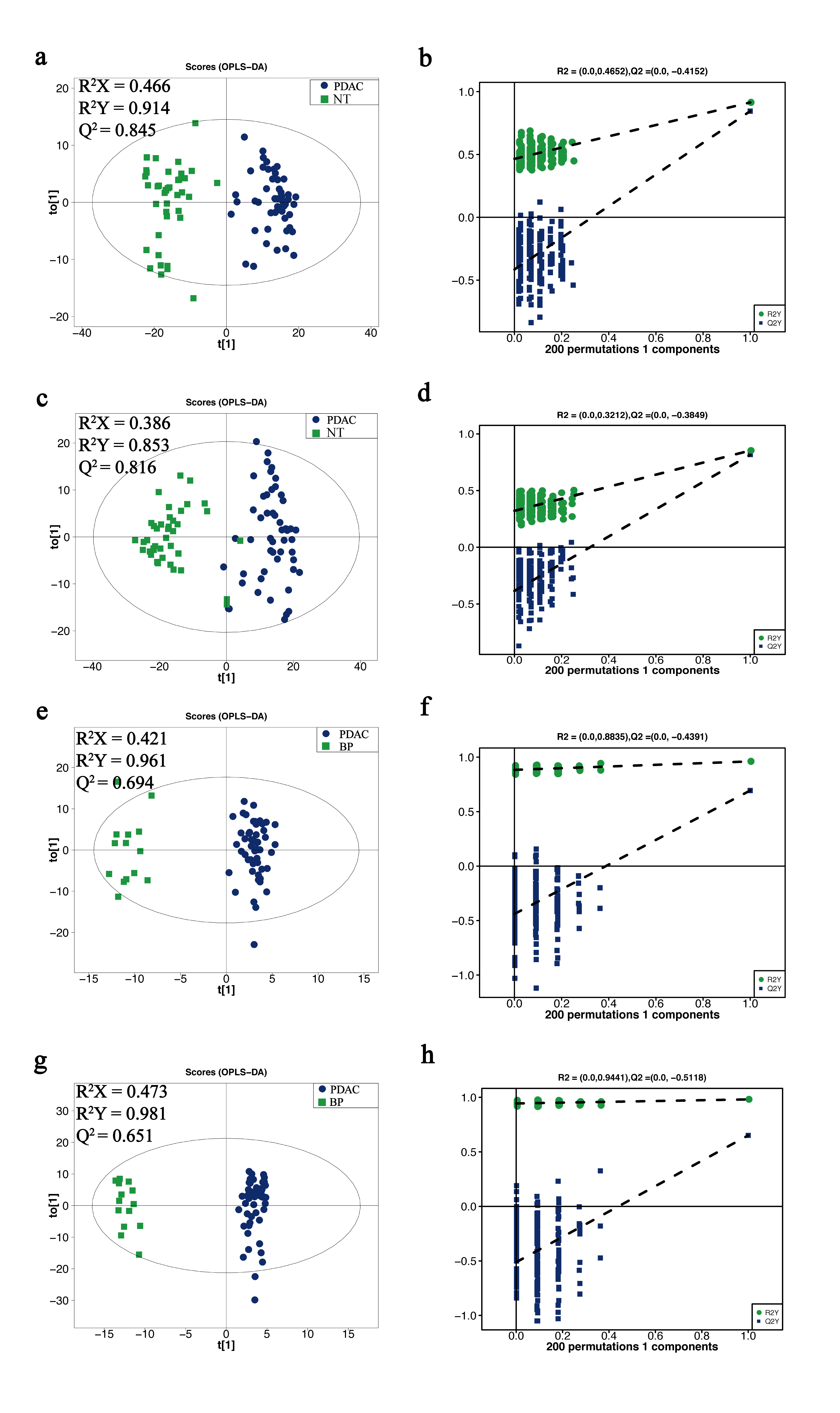


**Fig. S2 OPLS-DA score plot using tissue metabolomics analysis.** (A, C) OPLS-DA score plot of the comparison between PDAC and Ctr in positive mode (A) and negative mode (C). (E, G) OPLS-DA score plot of the comparison between PDAC and BP in positive mode (E) and negative mode (G). (B, D, F, H) The permutation tests show that these models have not been overfitted.


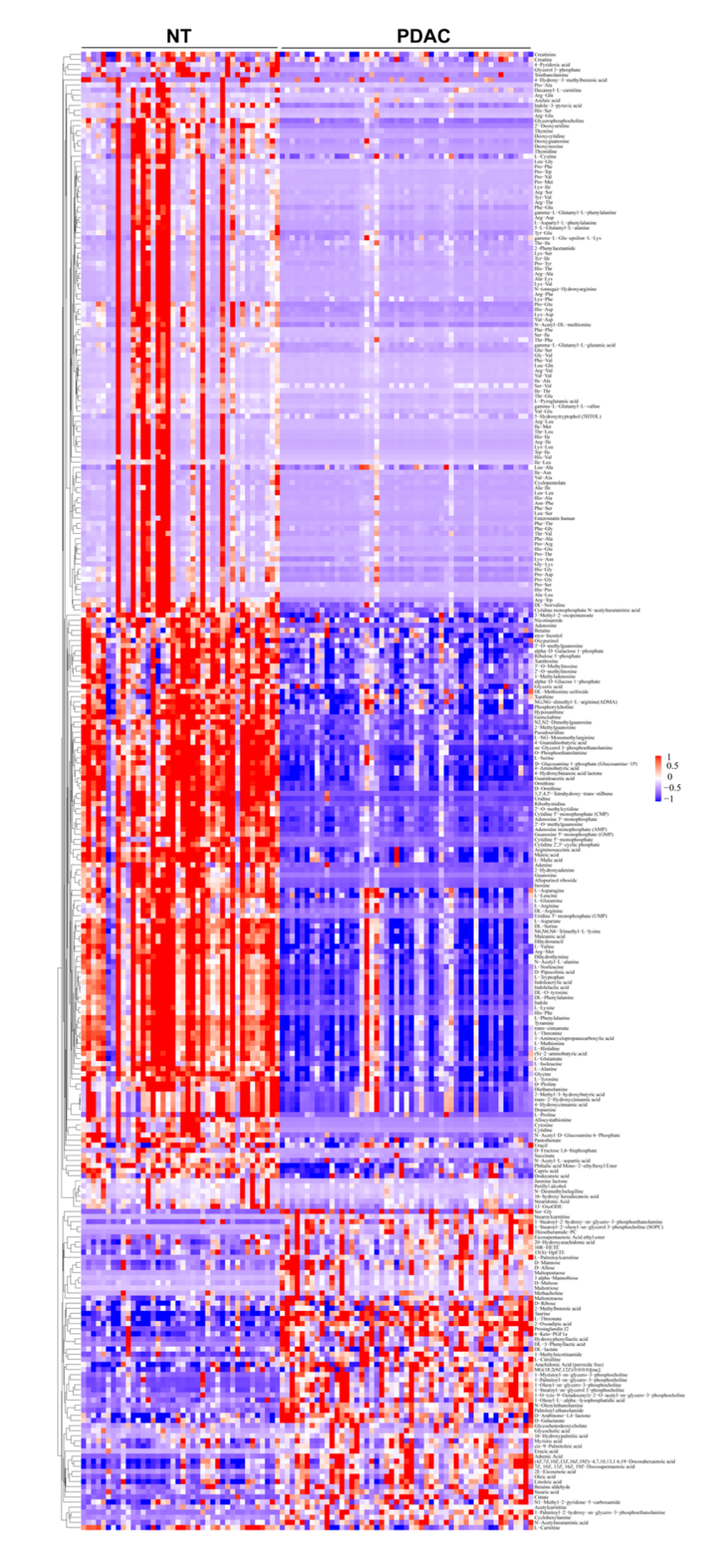


**Fig. S3. Heatmap of the 287 differential metabolites between PDAC tissues and noncancerous tissue (NT)**. It showed a significant discrimination between PDAC and NT.

**
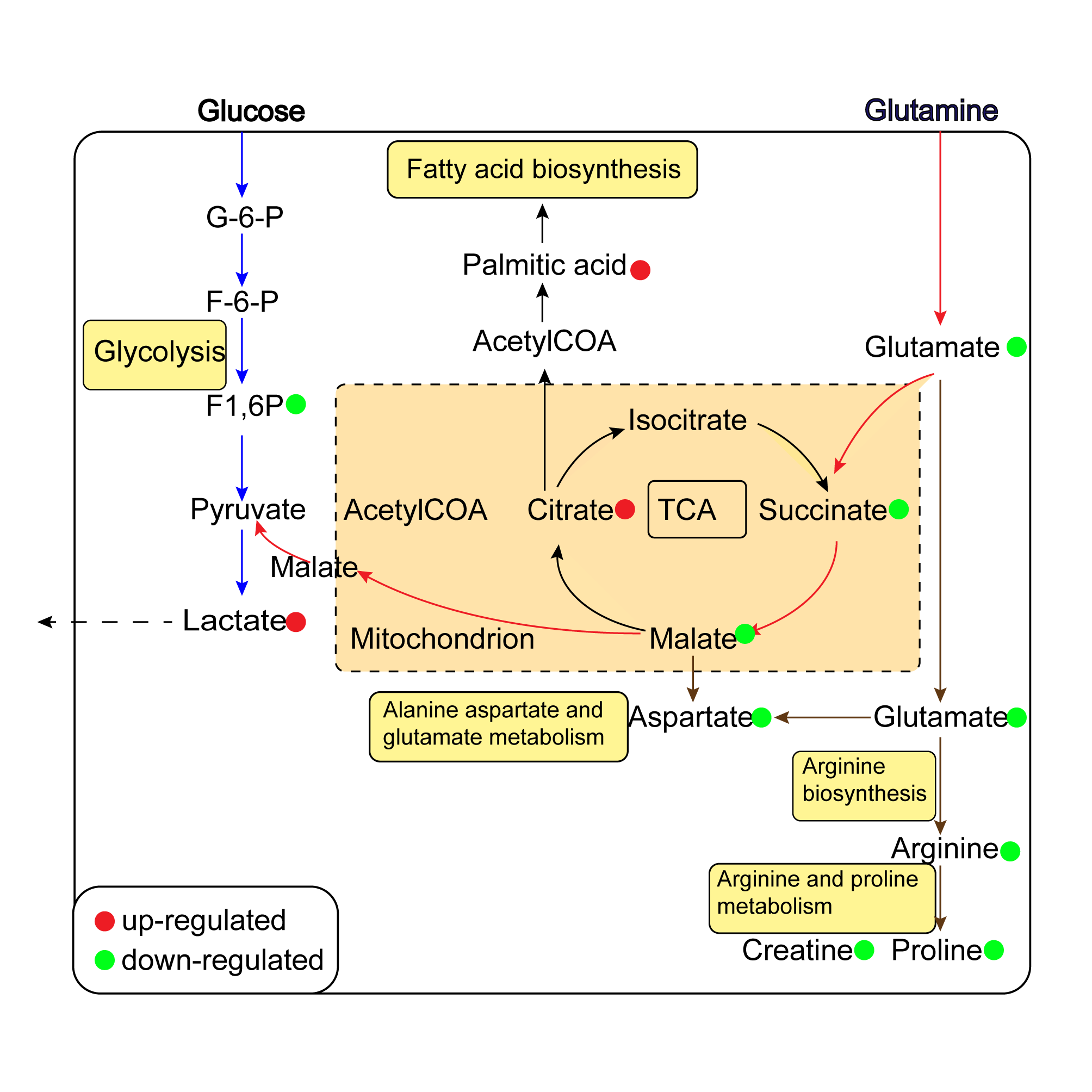
**

**Fig. S4. Perturbed metabolic pathways observed in PDAC.** The principal metabolic alterations of PDAC are the upregulated levels of fatty acids and downregulated levels of multiple amino acids and nucleotides. G-6-P, glucose-6-phosphate; F-6-P, fructose-6-phosphate; F-1,6-P, fructose 1,6-bisphosphate; TCA, tricarboxylic acid cycle.


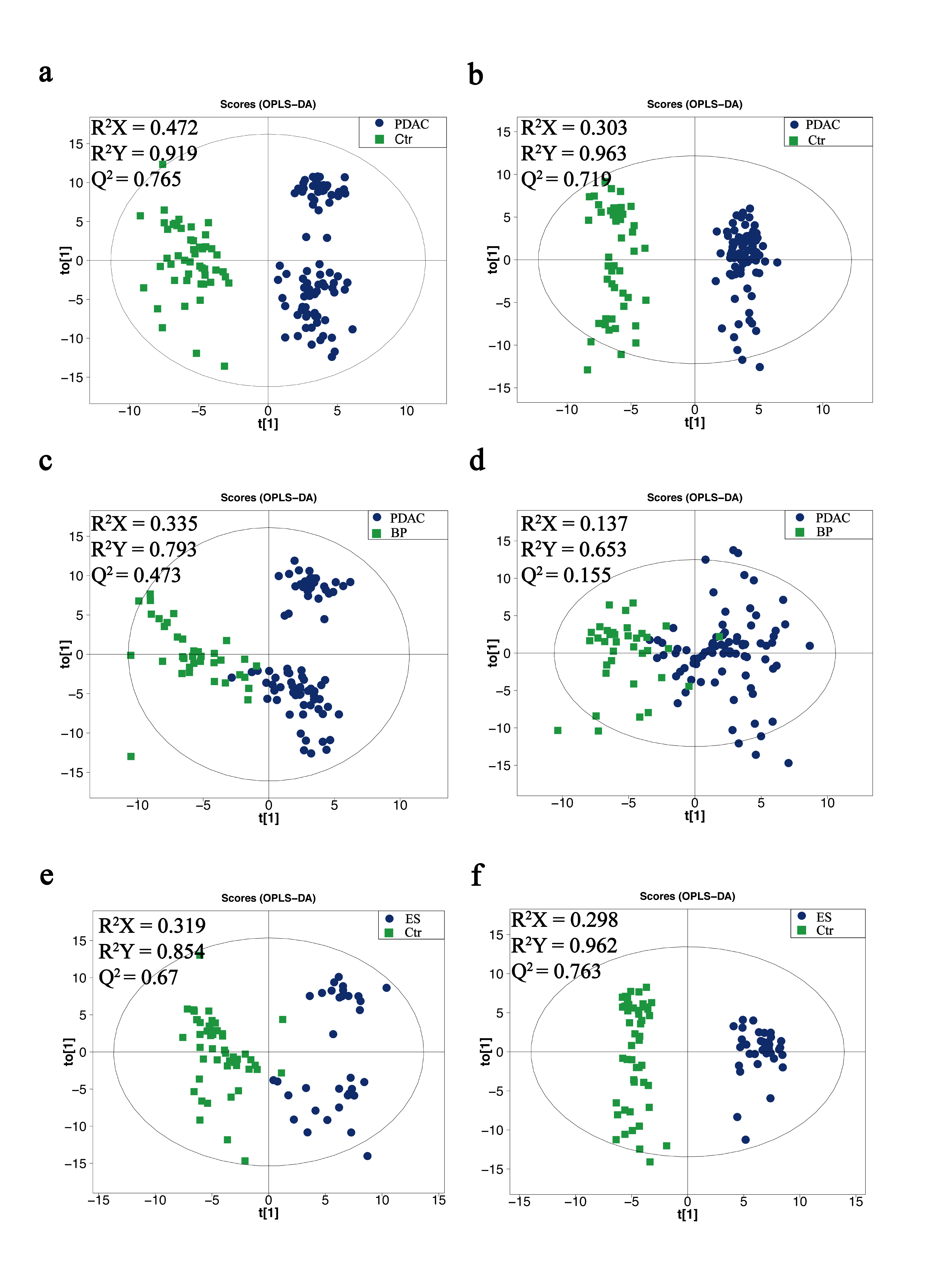


**Fig. S5 OPLS-DA score plot using serum metabolomics analysis.** (A, B) OPLS-DA score plot of the comparison between PDAC and Ctr in positive mode (A) and negative mode (B). (C, D) OPLS-DA score plot of the comparison between PDAC and BP in positive mode (C) and negative mode (D). (E, F) OPLS-DA score plot of the comparison between early-stage PDAC (ES) and Ctr (E, positive mode, F, negative mode)

**
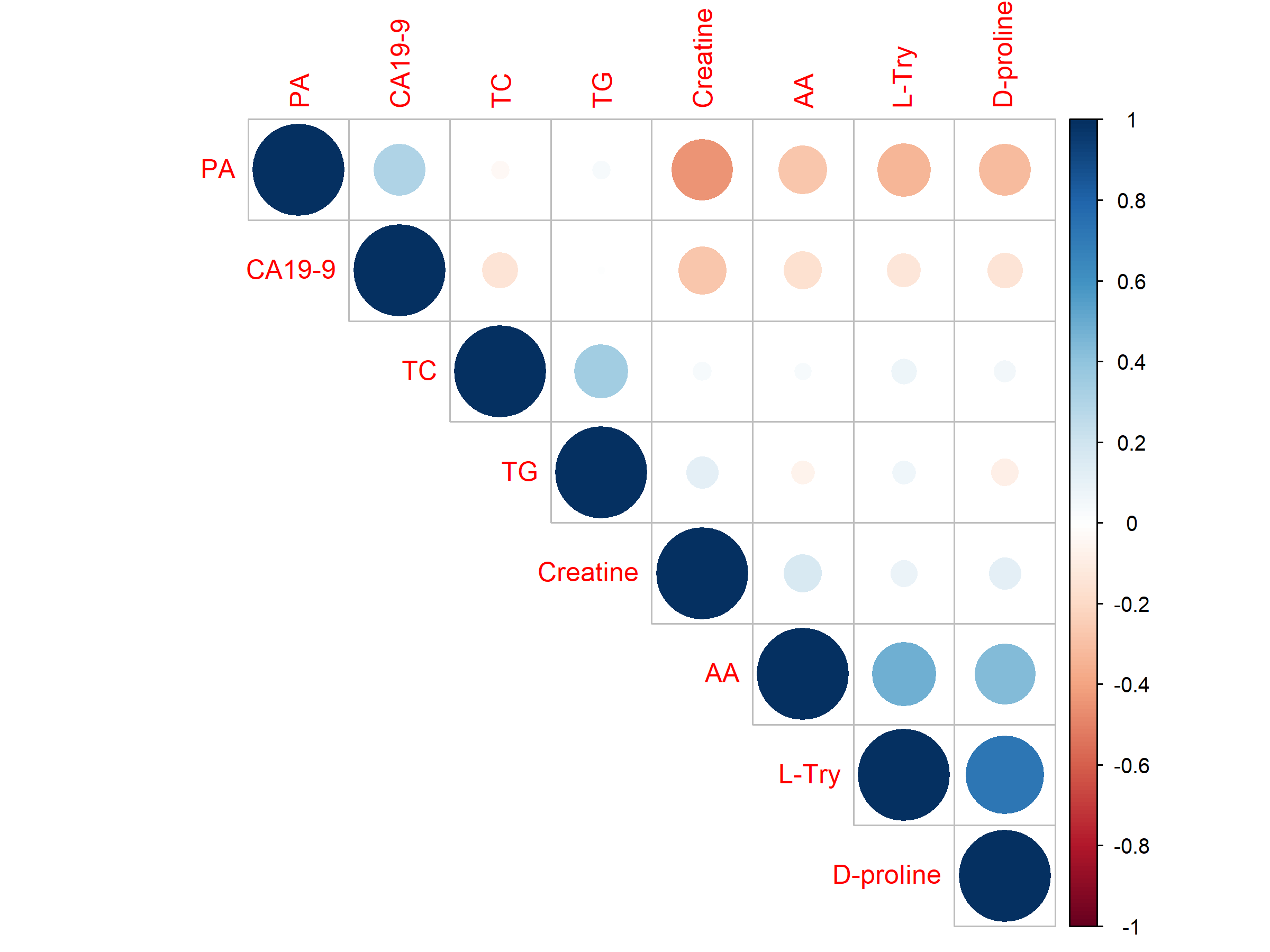
**

**Fig. S6 Correlation analysis between the concentrations of the 5 potential biomarkers and CA19-9, TC and TG in PDAC patients in the training set**. The red color represents negative correlation and blue color represents positive correlation. Circle size represents r value of metabolites and clinical characteristics. Abbreviations: CA19-9, carbohydrate antigen 19-9; TG, Triglyceride; TC, Total cholesterol; PA, palmitic acid; AA, arachidonic acid; L-Try, L-tryptophan.


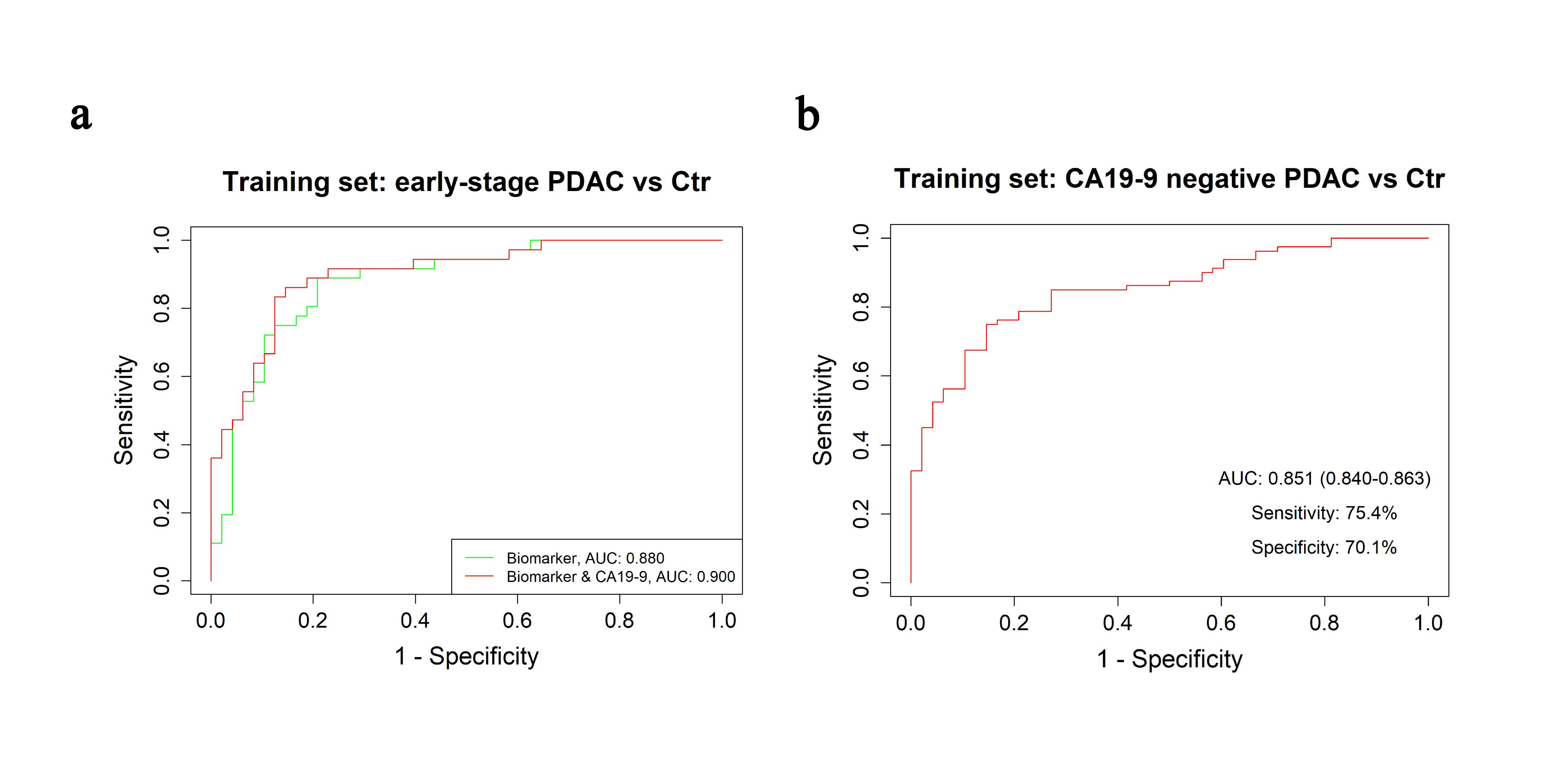


**Fig. S7 Diagnostic value of the biomarker panel**. (A) ROC curves of the biomarker panel and its combination with CA19-9 in the comparison of early-stage PDAC (n = 36) and Ctr in the training set. (B) ROC curves of the biomarker panel in the comparison of CA19-9 negative (CA19-9 < 37.0 IU/ml) PDAC (n = 20) and Ctr in the training set.

**Supplementary tables**

**Table S1. Major method information on LC-MS/MS quantitative analysis of amino acids in human serum**

| **Component Name** | **Analyte Mass Range** | **Analyte Retention Time (min)** | **Linear equation** | **R^2^** |
| --- | --- | --- | --- | --- |
| Glutamate | 148.1 / 84.1 | 8.773 | y = 0.029 x + -0.033 | 0.999 |
| Isoleucine | 132.1 / 86.2 | 4.188 | y = 0.025 x + -0.015 | 0.999 |
| Proline | 116.1 / 70.1 | 6.350 | y = 0.015 x + 0.024 | 0.998 |
| Creatine | 132.1 / 90.0 | 7.903 | y = 0.001 x + 0.002 | 0.999 |
| Glutamine | 147.0 / 84.1 | 8.386 | y = 0.032 x + -0.008 | 0.999 |
| Tryptophan | 205.1 / 146.3 | 3.709 | y = 11.547 x + -6.418 | 0.999 |
| Taurine | 126.0 / 44.1 | 7.118 | y = 0.002 x + 0.039 | 0.999 |

**Table S2. Major method information on GC-MS quantitative analysis of fatty acids in human serum**

| **Component Name** | **Abbreviation** | **Linear range (*ug/ml*)** | **Linear equation** | **R^2^** |
| --- | --- | --- | --- | --- |
| Myristic acid | C14:0 | 0.002-50 | y = 1.199 x - 0.002 | 0.999 |
| Palmitic acid | C16:0 | 0.004-100 | y = 1.018 x + 0.224 | 0.998 |
| Linoleic acid | C18:2N6 | 0.002-50 | y = 0.385 x - 8.394E-004 | 0.999 |
| Arachidonic Acid | C20:4N6 | 0.002-50 | y = 0.382 x - 0.008 | 0.999 |
| Adrenic Acid | C22:4N6 | 0.002-50 | y = 0.378 x - 0.023 | 0.999 |

**Table S3. Identified differential metabolites between PDAC tissue and noncancerous pancreatic tissue (NT)**

| **Metabolite** | **VIP** | **Fold change**^a^ | ***p*-value** | **FDR** | ***m/z*** | ***rt(s)*** | **Class** |
| --- | --- | --- | --- | --- | --- | --- | --- |
| Cytidine monophosphate N-acetylneuraminic acid | 1.14 | 0.16 | < 0.001 | < 0.001 | 653.11 | 509.06 | Pyrimidine nucleotides |
| 2'-O-methylinosine | 1.21 | 0.29 | < 0.001 | < 0.001 | 565.20 | 132.82 | Nucleosides and nucleotides |
| Xanthine | 2.07 | 0.61 | < 0.001 | < 0.001 | 153.04 | 195.90 | Alkaloids |
| alpha-D-Glucose 1-phosphate | 1.84 | 0.31 | < 0.001 | < 0.001 | 243.03 | 509.15 | Organooxygen compounds |
| Indole-3-pyruvic acid | 1.21 | 0.15 | < 0.001 | < 0.001 | 221.09 | 282.43 | Indoles and derivatives |
| Palmitoyl ethanolamide | 1.07 | 4.92 | < 0.001 | < 0.001 | 300.29 | 36.83 | Lipids |
| Phthalic acid Mono-2-ethylhexyl Ester | 1.11 | 0.48 | < 0.001 | < 0.001 | 296.19 | 32.19 | Carboxylic acids |
| N-Oleoylethanolamine | 1.24 | 6.98 | < 0.001 | < 0.001 | 326.30 | 36.04 | Organonitrogen compounds |
| 1-Myristoyl-sn-glycero-3-PC | 1.81 | 4.71 | < 0.001 | < 0.001 | 468.31 | 193.82 | Phospholipids |
| 1-Oleoyl-L-.alpha.-lysophosphatidic acid | 1.14 | 2.59 | < 0.001 | < 0.001 | 419.25 | 46.01 | Phospholipids |
| Jasmine lactone | 1.56 | 0.12 | 0.002 | 0.002 | 229.14 | 38.57 | Lactone |
| Val-Val | 1.76 | 0.04 | 0.003 | 0.003 | 217.15 | 199.29 | Dipeptides |
| His-Val | 1.79 | 0.04 | 0.003 | 0.004 | 255.14 | 318.99 | Dipeptides |
| Stearidonic Acid | 2.09 | 0.36 | 0.004 | 0.004 | 277.21 | 41.80 | Fatty Acids |
| Stearoylcarnitine | 2.17 | 35.03 | 0.004 | 0.005 | 428.37 | 154.22 | Fatty Acids |
| Perillyl alcohol | 1.14 | 0.16 | 0.014 | 0.015 | 213.15 | 81.51 | Hydrocarbons |
| N-Desmethylselegiline | 1.19 | 0.11 | 0.016 | 0.017 | 173.12 | 85.17 | Alkaloids |
| 16-hydroxy hexadecanoic acid | 1.33 | 0.24 | 0.027 | 0.028 | 295.23 | 80.36 | Fatty Acids |
| Glycochenodeoxycholate | 1.24 | 2.56 | 0.027 | 0.028 | 450.32 | 200.29 | Steroids and steroid derivatives |
| Eicosapentaenoic Acid ethyl ester | 1.11 | 1.73 | 0.048 | 0.048 | 331.26 | 41.40 | Fatty Acids |
| Guanidoacetic acid | 8.15 | 0.15 | < 0.001 | < 0.001 | 118.06 | 363.60 | Carboxylic acids and derivatives |
| Uridine | 6.39 | 0.07 | < 0.001 | < 0.001 | 489.15 | 148.59 | Pyrimidine nucleosides |
| D-Ornithine | 1.75 | 0.14 | < 0.001 | < 0.001 | 115.09 | 518.37 | Carboxylic acids and derivatives |
| sn-Glycerol 3-PE | 6.44 | 0.10 | < 0.001 | < 0.001 | 216.06 | 406.43 | Phospholipids |
| 4-Aminobutyric acid | 2.86 | 0.23 | < 0.001 | < 0.001 | 104.07 | 375.40 | Carboxylic acids and derivatives |
| Ornithine | 3.19 | 0.14 | < 0.001 | < 0.001 | 133.10 | 518.39 | Carboxylic acids and derivatives |
| 4-Hydroxybutanoic acid lactone | 2.45 | 0.24 | < 0.001 | < 0.001 | 87.04 | 375.39 | Lactones |
| 2'-O-methylguanosine | 2.70 | 0.11 | < 0.001 | < 0.001 | 364.06 | 485.92 | Nucleosides and nucleotides |
| Taurine | 10.06 | 2.84 | < 0.001 | < 0.001 | 126.02 | 289.09 | Amino acids |
| L-Valine | 4.65 | 0.39 | < 0.001 | < 0.001 | 118.09 | 292.89 | Amino acids |
| Arg-Met | 1.07 | 0.18 | < 0.001 | < 0.001 | 328.15 | 455.88 | Dipeptides |
| L-NG-Monomethylarginine | 1.20 | 0.23 | < 0.001 | < 0.001 | 189.13 | 525.37 | Amino acids |
| Pseudouridine | 1.01 | 0.25 | < 0.001 | < 0.001 | 245.08 | 226.19 | Nucleoside and nucleotides |
| L-Phenylalanine | 6.36 | 0.46 | < 0.001 | < 0.001 | 166.09 | 252.04 | Amino acids |
| D-Pipecolinic acid | 8.92 | 0.29 | < 0.001 | < 0.001 | 130.09 | 538.99 | Carboxylic acids |
| Adenosine 3'-monophosphate | 4.02 | 0.23 | < 0.001 | < 0.001 | 348.07 | 441.41 | Nucleoside and nucleotides |
| Gemcitabine | 1.03 | 0.14 | < 0.001 | < 0.001 | 549.13 | 428.19 |  |
| N2,N2-Dimethylguanosine | 1.51 | 0.20 | < 0.001 | < 0.001 | 312.13 | 177.11 | Purine nucleosides |
| N6,N6,N6-Trimethyl-L-lysine | 3.23 | 0.32 | < 0.001 | < 0.001 | 189.16 | 534.91 | Amino acids |
| L-Serine | 1.04 | 0.23 | < 0.001 | < 0.001 | 106.05 | 451.78 | Amino acids |
| Inosine | 5.98 | 0.09 | < 0.001 | < 0.001 | 269.09 | 200.58 | Purine nucleosides |
| L-Alanine | 1.17 | 0.44 | < 0.001 | < 0.001 | 90.05 | 348.13 | Amino acids |
| 1-Aminocyclopropanecarboxylic acid | 1.39 | 0.40 | < 0.001 | < 0.001 | 102.05 | 353.63 | Amino acids |
| 2-Methylguanosine | 2.53 | 0.20 | < 0.001 | < 0.001 | 298.11 | 183.80 | Purine nucleosides |
| L-Threonine | 2.90 | 0.38 | < 0.001 | < 0.001 | 120.06 | 353.52 | Amino acids |
| Tyramine | 6.36 | 0.53 | < 0.001 | < 0.001 | 120.08 | 252.04 | Amines |
| Allopurinol riboside | 6.08 | 0.05 | < 0.001 | < 0.001 | 537.17 | 200.58 | Nucleoside and nucleotides |
| 4-Guanidinobutyric acid | 2.88 | 0.13 | < 0.001 | < 0.001 | 146.09 | 367.21 | Amino acids |
| L-Methionine | 5.33 | 0.37 | < 0.001 | < 0.001 | 150.06 | 279.33 | Amino acids |
| 2'-O-methylcytidine | 2.68 | 0.14 | < 0.001 | < 0.001 | 324.06 | 473.23 | Nucleosides and nucleotides |
| Cytidine 2',3'-cyclic phosphate | 1.01 | 0.06 | < 0.001 | < 0.001 | 611.09 | 466.99 | Pyrimidine nucleotides |
| Indoleacrylic acid | 1.10 | 0.33 | < 0.001 | < 0.001 | 170.06 | 259.69 | Indoles and derivatives |
| 2-Hydroxyadenine | 4.81 | 0.07 | < 0.001 | < 0.001 | 152.06 | 255.41 | Alkaloids |
| DL-O-tyrosine | 2.75 | 0.33 | < 0.001 | < 0.001 | 146.06 | 260.94 | Amino acids |
| Indolelactic acid | 9.88 | 0.32 | < 0.001 | < 0.001 | 188.07 | 260.93 | Indoles and derivatives |
| Argininosuccinic acid | 3.99 | 0.23 | < 0.001 | < 0.001 | 291.13 | 514.73 | Carboxylic acids and derivatives |
| L-Norleucine | 4.78 | 0.19 | < 0.001 | < 0.001 | 263.20 | 258.37 | Amino acids |
| L-Aspartate | 1.39 | 0.28 | < 0.001 | < 0.001 | 267.08 | 433.14 | Amino acids s |
| Hypoxanthine | 8.11 | 0.60 | < 0.001 | < 0.001 | 137.05 | 155.59 | Alkaloids |
| DL-Phenylalanine | 4.98 | 0.16 | < 0.001 | < 0.001 | 331.16 | 252.07 | Amino acids |
| 1-Methyladenosine | 2.82 | 0.36 | < 0.001 | < 0.001 | 282.12 | 276.29 | Purine nucleosides |
| Adenosine | 3.18 | 0.10 | < 0.001 | < 0.001 | 250.09 | 87.94 | Purine nucleosides |
| L-Tryptophan | 7.11 | 0.25 | < 0.001 | < 0.001 | 205.10 | 260.93 | Amino acids |
| Adenine | 1.26 | 0.13 | < 0.001 | < 0.001 | 136.06 | 148.78 | Nucleosides and nucleotides |
| Phosphorylcholine | 8.60 | 0.44 | < 0.001 | < 0.001 | 184.07 | 514.59 | Phospholipids |
| L-Lysine | 2.80 | 0.20 | < 0.001 | < 0.001 | 293.22 | 538.79 | Amino acids |
| Cytidine 5'-monophosphate | 2.26 | 0.03 | < 0.001 | < 0.001 | 306.05 | 331.71 | Nucleosides and nucleotides |
| L-Leucine | 1.34 | 0.61 | < 0.001 | < 0.001 | 132.10 | 259.63 | Amino acids |
| L-Glutamate | 1.72 | 0.39 | < 0.001 | < 0.001 | 295.11 | 423.88 | Amino acids |
| N-Acetyl-D-Glucosamine 6-Phosphate | 1.50 | 0.12 | < 0.001 | < 0.001 | 284.05 | 491.96 | Carbohydrates |
| L-Glutamine | 1.29 | 0.26 | < 0.001 | < 0.001 | 293.14 | 383.64 | Amino acids |
| His-Phe | 1.62 | 0.17 | < 0.001 | < 0.001 | 302.14 | 384.30 | Dipeptides |
| 3'-O-Methylinosine | 1.58 | 0.44 | < 0.001 | < 0.001 | 283.10 | 132.56 | Nucleosides and nucleotides |
| L-Asparagine | 1.22 | 0.54 | < 0.001 | < 0.001 | 133.06 | 389.03 | Amino acids |
| Glycerophosphocholine | 1.39 | 0.08 | < 0.001 | < 0.001 | 258.11 | 459.91 | Phospholipids |
| DL-Norvaline | 1.82 | 0.18 | < 0.001 | < 0.001 | 235.16 | 292.31 | Amino acids |
| D-Proline | 2.86 | 0.26 | < 0.001 | < 0.001 | 116.07 | 523.40 | Amino acids |
| NG,NG-dimethyl-L-arginine(ADMA) | 4.52 | 0.44 | < 0.001 | < 0.001 | 203.15 | 491.94 | Amino acids |
| Guanosine | 1.36 | 0.03 | < 0.001 | < 0.001 | 567.19 | 255.51 | Purine nucleosides |
| Diethanolamine | 1.91 | 0.37 | < 0.001 | < 0.001 | 70.06 | 524.61 | Amines |
| DL-Methionine sulfoxide | 1.12 | 0.37 | < 0.001 | < 0.001 | 166.05 | 379.52 | Amino acids |
| Dopamine | 3.16 | 0.35 | < 0.001 | < 0.001 | 136.08 | 304.85 | Amines |
| L-Proline | 2.36 | 0.12 | < 0.001 | < 0.001 | 231.13 | 300.23 | Amino acids |
| 4-Hydroxycinnamic acid | 1.21 | 0.36 | < 0.001 | < 0.001 | 147.04 | 304.90 | Carboxylic acids |
| 2'-Deoxyuridine | 1.59 | 0.02 | < 0.001 | < 0.001 | 457.15 | 104.20 | Pyrimidine nucleosides |
| trans-2-Hydroxycinnamic acid | 2.78 | 0.34 | < 0.001 | < 0.001 | 165.05 | 304.85 | Carboxylic acids |
| L-Arginine | 12.78 | 0.32 | < 0.001 | < 0.001 | 175.12 | 525.88 | Amino acids |
| 1-Oleoyl-sn-glycero-3-PC | 8.95 | 9.70 | < 0.001 | < 0.001 | 522.35 | 178.86 | Phospholipids |
| 2-Methyl-3-hydroxybutyric acid | 3.26 | 0.32 | < 0.001 | < 0.001 | 182.08 | 304.85 | Fatty Acids |
| Allocystathionine | 1.52 | 0.03 | < 0.001 | < 0.001 | 223.07 | 490.94 | Amino acids |
| Nicotinamide | 3.64 | 0.66 | < 0.001 | < 0.001 | 123.06 | 50.52 | Pyridines and derivatives |
| 1-Stearoyl-sn-glycerol 3-PC | 1.64 | 5.09 | < 0.001 | < 0.001 | 546.35 | 177.40 | Phospholipids |
| Pro-Glu | 2.71 | 0.03 | < 0.001 | < 0.001 | 245.11 | 452.95 | Dipeptides |
| Cytosine | 4.72 | 0.02 | < 0.001 | < 0.001 | 112.05 | 222.56 | Alkaloids |
| Prostaglandin I2 | 2.08 | 6.18 | < 0.001 | < 0.001 | 353.23 | 167.64 | Fatty Acids |
| Arachidonic Acid (peroxide free) | 2.31 | 2.00 | < 0.001 | < 0.001 | 322.27 | 38.84 | Fatty Acids |
| Arg-Trp | 1.15 | 0.05 | < 0.001 | < 0.001 | 343.20 | 298.81 | Dipeptides |
| Betaine | 3.27 | 0.62 | < 0.001 | < 0.001 | 118.09 | 260.77 | Carboxylic acids and derivatives |
| His-Asp | 1.30 | 0.10 | < 0.001 | < 0.001 | 271.10 | 459.78 | Dipeptides |
| DL-Arginine | 2.95 | 0.19 | < 0.001 | < 0.001 | 349.23 | 525.55 | Amino acids |
| 1-Methylnicotinamide | 4.00 | 3.22 | < 0.001 | < 0.001 | 137.07 | 286.53 | Pyridines and derivatives |
| Lys-Asn | 1.20 | 0.12 | < 0.001 | < 0.001 | 261.15 | 481.00 | Dipeptides |
| 1-Stearoyl-2-hydroxy-sn-glycero-3-PE | 2.34 | 5.48 | < 0.001 | < 0.001 | 482.32 | 182.88 | Phospholipids |
| His-Gly | 1.15 | 0.10 | < 0.001 | < 0.001 | 213.10 | 400.13 | Dipeptides |
| Cytidine | 5.18 | 0.01 | < 0.001 | < 0.001 | 487.18 | 222.28 | Pyrimidine nucleosides |
| Pro-Gly | 1.71 | 0.08 | < 0.001 | < 0.001 | 173.09 | 357.44 | Dipeptides |
| Tyr-Glu | 1.01 | 0.13 | < 0.001 | < 0.001 | 311.12 | 403.08 | Dipeptides |
| Lys-Asp | 1.67 | 0.07 | < 0.001 | < 0.001 | 262.14 | 492.83 | Dipeptides |
| D-Mannose | 1.97 | 2.88 | < 0.001 | < 0.001 | 198.10 | 274.36 | Carbohydrates |
| 1-Palmitoyl-2-hydroxy-sn-glycero-3-PE | 3.00 | 8.24 | < 0.001 | < 0.001 | 454.29 | 193.12 | Phospholipids |
| Ser-Gly | 2.35 | 5.80 | < 0.001 | < 0.001 | 163.07 | 356.75 | Dipeptides |
| Pro-Asp | 1.45 | 0.09 | < 0.001 | < 0.001 | 231.10 | 461.38 | Dipeptides |
| 1-Palmitoyl-sn-glycero-3-PC | 13.42 | 3.97 | < 0.001 | < 0.001 | 496.34 | 194.50 | Phospholipids |
| Thymine | 2.48 | 0.03 | < 0.001 | < 0.001 | 127.05 | 88.04 | Alkaloids |
| 1-O-(cis-9-Octadecenyl)-2-O-acetyl-sn-glycero-3-PC | 1.54 | 7.32 | < 0.001 | < 0.001 | 550.38 | 175.01 | Phospholipids |
| Triethanolamine | 4.27 | 0.18 | < 0.001 | < 0.001 | 150.11 | 121.98 | Amines |
| Deoxyinosine | 1.55 | 0.04 | < 0.001 | < 0.001 | 253.09 | 164.79 | Purine nucleosides |
| Enterostatin human | 1.66 | 0.02 | < 0.001 | < 0.001 | 497.28 | 468.23 | Peptides |
| L-Cystine | 1.58 | 0.35 | < 0.001 | < 0.001 | 241.03 | 468.52 | Amino acids |
| Glu-Ser | 1.32 | 0.07 | < 0.001 | < 0.001 | 235.09 | 457.48 | Dipeptides |
| Deoxyguanosine | 1.24 | 0.13 | < 0.001 | < 0.001 | 268.10 | 220.83 | Purine nucleosides |
| Phe-Glu | 1.54 | 0.13 | < 0.001 | < 0.001 | 295.13 | 366.31 | Dipeptides |
| 5-L-Glutamyl-L-alanine | 1.60 | 0.03 | < 0.001 | < 0.001 | 201.09 | 438.21 | Amino acids |
| Deoxycytidine | 1.30 | 0.02 | < 0.001 | < 0.001 | 228.10 | 191.01 | Pyrimidine nucleosides |
| Val-Asp | 1.58 | 0.02 | < 0.001 | < 0.001 | 233.11 | 402.48 | Dipeptides |
| L-Aspartyl-L-phenylalanine | 1.50 | 0.06 | < 0.001 | < 0.001 | 281.11 | 368.42 | Dipeptides |
| Pro-Tyr | 1.27 | 0.07 | < 0.001 | < 0.001 | 279.13 | 276.07 | Dipeptides |
| Ala-Ile | 1.30 | 0.04 | < 0.001 | < 0.001 | 185.13 | 242.17 | Dipeptides |
| gamma-L-Glutamyl-L-glutamic acid | 1.46 | 0.15 | < 0.001 | < 0.001 | 277.10 | 510.52 | Amino acids |
| Glycerol 3-phosphate | 1.46 | 0.03 | < 0.001 | < 0.001 | 345.03 | 461.64 | Glycerophospholipids |
| Val-Ala | 2.06 | 0.05 | < 0.001 | < 0.001 | 171.11 | 213.79 | Dipeptides |
| L-Palmitoylcarnitine | 2.39 | 18.18 | < 0.001 | < 0.001 | 400.34 | 178.08 | Fatty Acids |
| Leu-Leu | 1.68 | 0.02 | < 0.001 | < 0.001 | 227.17 | 190.51 | Dipeptides |
| Cyclopentolate | 1.01 | 0.08 | < 0.001 | < 0.001 | 333.21 | 498.33 | Carboxylic acids |
| Thr-Val | 1.47 | 0.09 | < 0.001 | < 0.001 | 201.12 | 202.98 | Dipeptides |
| His-Glu | 2.31 | 0.08 | < 0.001 | < 0.001 | 285.12 | 459.11 | Dipeptides |
| Cyclohexylamine | 2.86 | 8.07 | < 0.001 | < 0.001 | 160.13 | 393.46 | Amines |
| Phe-Gly | 1.26 | 0.14 | < 0.001 | < 0.001 | 223.11 | 224.86 | Dipeptides |
| Arg-Ile | 3.24 | 0.02 | < 0.001 | < 0.001 | 288.20 | 360.08 | Dipeptides |
| MG(18:2(9Z,12Z)/0:0/0:0)[rac] | 1.01 | 2.11 | < 0.001 | < 0.001 | 337.27 | 75.40 |  |
| Lys-Val | 3.07 | 0.02 | < 0.001 | < 0.001 | 246.18 | 401.84 | Dipeptides |
| N-(omega)-Hydroxyarginine | 1.43 | 0.06 | < 0.001 | < 0.001 | 232.14 | 454.97 | Amino acids |
| Leu-Ser | 2.49 | 0.04 | < 0.001 | < 0.001 | 260.16 | 261.68 | Dipeptides |
| Arg-Asp | 1.32 | 0.05 | < 0.001 | < 0.001 | 290.14 | 484.87 | Dipeptides |
| Phe-Ser | 1.53 | 0.04 | < 0.001 | < 0.001 | 294.14 | 258.51 | Dipeptides |
| 2-Phenylacetamide | 2.31 | 0.12 | < 0.001 | < 0.001 | 196.10 | 42.84 | Carboxylic acids |
| Pro-Arg | 1.65 | 0.04 | < 0.001 | < 0.001 | 272.17 | 454.24 | Dipeptides |
| His-Ala | 1.87 | 0.06 | < 0.001 | < 0.001 | 227.11 | 357.89 | Dipeptides |
| Tyr-Ile | 2.92 | 0.03 | < 0.001 | < 0.001 | 295.16 | 195.91 | Dipeptides |
| His-Thr | 1.34 | 0.04 | < 0.001 | < 0.001 | 257.12 | 357.92 | Dipeptides |
| Ala-Lys | 2.15 | 0.05 | < 0.001 | < 0.001 | 218.15 | 447.73 | Dipeptides |
| Lys-Leu | 3.15 | 0.02 | < 0.001 | < 0.001 | 260.20 | 373.60 | Dipeptides |
| Arg-Ala | 1.93 | 0.05 | < 0.001 | < 0.001 | 246.15 | 430.17 | Dipeptides |
| His-Ile | 4.61 | 0.01 | < 0.001 | < 0.001 | 269.16 | 293.64 | Dipeptides |
| Lys-Ser | 1.09 | 0.08 | < 0.001 | < 0.001 | 234.14 | 457.05 | Dipeptides |
| Pro-Thr | 2.03 | 0.04 | < 0.001 | < 0.001 | 217.12 | 326.90 | Dipeptides |
| Asn-Phe | 1.68 | 0.08 | < 0.001 | < 0.001 | 280.13 | 260.20 | Dipeptides |
| Phe-Ala | 1.21 | 0.04 | < 0.001 | < 0.001 | 237.12 | 197.25 | Dipeptides |
| Pro-Met | 1.11 | 0.03 | < 0.001 | < 0.001 | 247.11 | 267.39 | Dipeptides |
| Phe-Thr | 1.10 | 0.06 | < 0.001 | < 0.001 | 267.13 | 197.71 | Dipeptides |
| L-Citrulline | 2.23 | 2.68 | < 0.001 | < 0.001 | 176.10 | 405.96 | Amino acids |
| Pro-Trp | 1.07 | 0.04 | < 0.001 | < 0.001 | 302.15 | 247.08 | Dipeptides |
| .gamma.-L-Glu-.epsilon.-L-Lys | 2.90 | 0.24 | < 0.001 | < 0.001 | 276.15 | 505.75 | Dipeptides |
| Leu-Ala | 1.05 | 0.39 | < 0.001 | < 0.001 | 203.14 | 41.51 | Dipeptides |
| Leu-Gly | 1.89 | 0.06 | < 0.001 | < 0.001 | 189.12 | 276.85 | Dipeptides |
| Pro-Val | 2.99 | 0.02 | < 0.001 | < 0.001 | 215.14 | 275.73 | Dipeptides |
| Gly-Lys | 1.32 | 0.06 | < 0.001 | 0.001 | 204.13 | 474.14 | Dipeptides |
| Thioetheramide-PC | 3.09 | 45.27 | < 0.001 | 0.001 | 758.57 | 46.66 | Phospholipids |
| Maltotetraose | 1.69 | 2.33 | < 0.001 | 0.001 | 667.23 | 530.93 | Carbohydrates |
| Ile-Asn | 1.28 | 0.07 | < 0.001 | 0.001 | 228.13 | 223.43 | Dipeptides |
| Arg-Thr | 1.17 | 0.06 | < 0.001 | 0.001 | 276.16 | 417.05 | Dipeptides |
| Thr-Leu | 2.42 | 0.02 | < 0.001 | 0.001 | 233.15 | 229.70 | Dipeptides |
| Arg-Ser | 1.20 | 0.09 | < 0.001 | 0.001 | 262.15 | 440.63 | Dipeptides |
| Arg-Phe | 1.01 | 0.07 | < 0.001 | 0.001 | 322.19 | 347.90 | Dipeptides |
| Thr-Glu | 1.12 | 0.08 | < 0.001 | 0.001 | 249.11 | 446.58 | Dipeptides |
| Lys-Phe | 1.10 | 0.11 | 0.001 | 0.001 | 294.18 | 361.44 | Dipeptides |
| N-Acetylneuraminic acid | 1.72 | 2.15 | 0.001 | 0.001 | 310.11 | 381.77 | Carbohydrates |
| Val-Glu | 1.07 | 0.12 | 0.001 | 0.001 | 247.13 | 400.84 | Dipeptides |
| Uracil | 4.48 | 0.82 | 0.001 | 0.001 | 113.03 | 80.47 | Diazines |
| Ala-Leu | 1.25 | 0.06 | 0.001 | 0.001 | 185.13 | 199.99 | Dipeptides |
| Trp-Ile | 2.24 | 0.01 | 0.001 | 0.001 | 318.18 | 179.52 | Dipeptides |
| Thr-Ile | 1.28 | 0.17 | 0.001 | 0.001 | 274.17 | 45.99 | Dipeptides |
| Lys-Ile | 1.49 | 0.06 | 0.001 | 0.001 | 260.20 | 356.74 | Dipeptides |
| N1-Methyl-2-pyridone-5-carboxamide | 1.08 | 1.51 | 0.001 | 0.001 | 153.06 | 70.80 | Pyridines and derivatives |
| His-Pro | 1.81 | 0.04 | 0.001 | 0.001 | 235.12 | 128.28 | Dipeptides |
| Tyr-Val | 1.75 | 0.07 | 0.001 | 0.001 | 281.15 | 206.58 | Dipeptides |
| Gly-Val | 1.00 | 0.04 | 0.001 | 0.001 | 175.11 | 275.36 | Dipeptides |
| L-Pyroglutamic acid | 1.36 | 0.04 | 0.001 | 0.001 | 259.09 | 509.03 | Amino acids |
| His-Ser | 1.72 | 0.06 | 0.001 | 0.001 | 284.13 | 377.70 | Dipeptides |
| Arg-Leu | 1.31 | 0.03 | 0.001 | 0.002 | 288.20 | 336.35 | Dipeptides |
| Ile-Ala | 2.69 | 0.02 | 0.001 | 0.002 | 203.14 | 217.14 | Dipeptides |
| Pro-Ser | 1.08 | 0.08 | 0.002 | 0.002 | 203.10 | 364.49 | Dipeptides |
| Phe-Val | 1.63 | 0.03 | 0.002 | 0.002 | 265.15 | 178.74 | Dipeptides |
| Ile-Met | 1.21 | 0.04 | 0.002 | 0.002 | 263.14 | 180.49 | Dipeptides |
| Betaine aldehyde | 1.05 | 2.22 | 0.002 | 0.002 | 102.09 | 277.00 | Amines |
| Ser-Ile | 1.16 | 0.04 | 0.002 | 0.002 | 219.13 | 248.05 | Dipeptides |
| Pro-Phe | 2.26 | 0.02 | 0.002 | 0.002 | 263.14 | 233.21 | Dipeptides |
| 5-Hydroxytryptophol (5HTOL) | 1.49 | 0.19 | 0.002 | 0.002 | 219.11 | 40.42 | Indoles and derivatives |
| Maltopentaose | 1.02 | 10.95 | 0.002 | 0.002 | 846.31 | 556.08 | Carbohydrates |
| Phe-Phe | 1.80 | 0.05 | 0.002 | 0.003 | 313.15 | 117.23 | Dipeptides |
| Leu-Gln | 2.07 | 0.03 | 0.003 | 0.003 | 260.16 | 280.72 | Dipeptides |
| Arg-Val | 1.26 | 0.06 | 0.003 | 0.003 | 274.19 | 363.02 | Dipeptides |
| 20-Hydroxyarachidonic acid | 2.33 | 1.80 | 0.003 | 0.003 | 303.23 | 42.72 | Fatty Acids |
| Pro-Ala | 1.27 | 0.02 | 0.004 | 0.005 | 187.11 | 330.58 | Dipeptides |
| 1-Stearoyl-2-oleoyl-sn-glycerol 3-PC (SOPC) | 2.14 | 31.20 | 0.004 | 0.005 | 810.60 | 46.31 | Phospholipids |
| Acetylcarnitine | 5.08 | 2.14 | 0.004 | 0.005 | 204.12 | 301.98 | Fatty Acids |
| Thr-Phe | 1.26 | 0.13 | 0.004 | 0.005 | 267.13 | 212.78 | Dipeptides |
| Arg-Glu | 2.07 | 0.06 | 0.006 | 0.007 | 364.18 | 238.95 | Dipeptides |
| Decanoyl-L-carnitine | 1.14 | 0.28 | 0.006 | 0.007 | 316.25 | 189.35 | Lipids |
| 3.alpha.-Mannobiose | 1.66 | 17.46 | 0.010 | 0.010 | 360.15 | 395.82 | Carbohydrate |
| Ile-Thr | 2.15 | 0.03 | 0.010 | 0.010 | 233.15 | 214.88 | Dipeptides |
| Arg-Gln | 2.00 | 0.07 | 0.010 | 0.011 | 302.17 | 86.66 | Dipeptides |
| Ile-Leu | 1.41 | 0.03 | 0.014 | 0.015 | 245.18 | 161.41 | Dipeptides |
| Creatinine | 2.11 | 0.82 | 0.018 | 0.019 | 114.07 | 158.11 | Amino acids |
| Azelaic acid | 2.09 | 0.17 | 0.019 | 0.020 | 171.10 | 42.31 | Fatty Acids |
| L-Carnitine | 7.82 | 0.84 | 0.020 | 0.021 | 162.11 | 353.39 | Lipids |
| Ser-Val | 1.10 | 0.19 | 0.027 | 0.028 | 205.12 | 281.17 | Dipeptides |
| Maltotriose | 1.09 | 21.61 | 0.038 | 0.038 | 522.20 | 469.05 | Carbohydrates |
| Creatine | 1.87 | 0.71 | 0.041 | 0.042 | 132.08 | 354.66 | Amino acids |
| O-PE | 1.24 | 0.13 | < 0.001 | < 0.001 | 140.01 | 406.55 | Phospholipids |
| D-Glucosamine 1-phosphate (Glucosamine-1P) | 3.11 | 0.16 | < 0.001 | < 0.001 | 258.04 | 446.55 | Glycolysis metabolites |
| DL-Serine | 2.92 | 0.43 | < 0.001 | < 0.001 | 104.04 | 386.03 | Amino acids |
| Ribothymidine | 3.64 | 0.07 | < 0.001 | < 0.001 | 257.08 | 131.71 | Pyrimidine nucleosides |
| Maleic acid | 1.63 | 0.53 | < 0.001 | < 0.001 | 115.00 | 428.52 | Tricarboxylic acid cycle metabolites |
| Adenosine monophosphate (AMP) | 2.69 | 0.21 | < 0.001 | < 0.001 | 346.05 | 439.54 | Purine nucleotides |
| Cytidine 5'-monophosphate (CMP) | 3.86 | 0.14 | < 0.001 | < 0.001 | 322.04 | 471.51 | Pyrimidine nucleotides |
| trans-cinnamate | 4.61 | 0.39 | < 0.001 | < 0.001 | 147.05 | 255.54 | Carboxylic acids |
| 3,3',4,5'-Tetrahydroxy-trans-stilbene | 10.57 | 0.02 | < 0.001 | < 0.001 | 487.13 | 148.13 | Hydrocarbons |
| L-Histidine | 6.17 | 0.25 | < 0.001 | < 0.001 | 154.06 | 386.23 | Amino acids |
| Xanthosine | 1.68 | 0.32 | < 0.001 | < 0.001 | 283.07 | 206.14 | Purine nucleosides |
| Guanosine 5'-monophosphate (GMP) | 2.69 | 0.11 | < 0.001 | < 0.001 | 362.05 | 480.69 | Fatty Acids |
| Maleamic acid | 1.14 | 0.37 | < 0.001 | < 0.001 | 114.02 | 388.92 | Carboxylic acids |
| Dihydrouracil | 1.80 | 0.33 | < 0.001 | < 0.001 | 113.04 | 388.93 | Diazines |
| Dihydrothymine | 2.44 | 0.35 | < 0.001 | < 0.001 | 127.05 | 384.33 | Diazines |
| alpha-D-Galactose 1-phosphate | 1.52 | 0.39 | < 0.001 | < 0.001 | 259.02 | 491.29 | Carbohydrates |
| 2-Oxoadipic acid | 10.71 | 1.89 | < 0.001 | < 0.001 | 141.02 | 345.79 | Keto acids and derivatives |
| Glycine | 2.20 | 0.49 | < 0.001 | < 0.001 | 74.03 | 371.13 | Amino acids |
| L-Tyrosine | 10.44 | 0.33 | < 0.001 | < 0.001 | 180.07 | 306.93 | Amino acids |
| L-Malic acid | 2.65 | 0.56 | < 0.001 | < 0.001 | 133.01 | 428.13 | Tricarboxylic acid cycle metabolites |
| Indole | 2.01 | 0.28 | < 0.001 | < 0.001 | 116.05 | 264.23 | Indoles and derivatives |
| N-Acetyl-L-alanine | 2.29 | 0.36 | < 0.001 | < 0.001 | 130.05 | 233.40 | Amino acids |
| (S)-2-aminobutyric acid | 2.24 | 0.52 | < 0.001 | < 0.001 | 102.06 | 420.64 | Amino acids |
| L-Isoleucine | 1.82 | 0.17 | < 0.001 | < 0.001 | 261.18 | 258.81 | Amino acids |
| Oxypurinol | 1.01 | 0.44 | < 0.001 | < 0.001 | 151.03 | 218.60 | Diazines |
| myo-Inositol | 2.57 | 0.52 | < 0.001 | < 0.001 | 179.06 | 385.05 | Hydrocarbons |
| 3-Methyl-2-oxopentanoate | 2.99 | 0.28 | < 0.001 | < 0.001 | 129.06 | 47.39 | Keto acids and derivatives |
| L-Threonate | 1.81 | 2.11 | < 0.001 | < 0.001 | 135.03 | 276.34 | Amino acids |
| Ribulose 5-phosphate | 1.11 | 0.41 | < 0.001 | < 0.001 | 289.03 | 501.16 | Nucleosides and nucleotides |
| Uridine 5'-monophosphate (UMP) | 2.56 | 0.06 | < 0.001 | < 0.001 | 323.03 | 446.89 | Pyrimidine nucleotides |
| 3'-O-methylguanosine | 2.04 | 0.36 | < 0.001 | < 0.001 | 296.10 | 188.91 | Purine nucleosides |
| Capric acid | 2.49 | 0.33 | < 0.001 | < 0.001 | 171.14 | 43.96 | Fatty Acids |
| Glyceric acid | 1.20 | 0.62 | < 0.001 | < 0.001 | 105.02 | 273.16 | Carbohydrates |
| 6-Keto-PGF1a | 2.08 | 2.62 | < 0.001 | < 0.001 | 369.23 | 118.55 | Lipids |
| Pantothenate | 1.63 | 0.44 | < 0.001 | < 0.001 | 218.10 | 253.43 | Vitamins |
| N-Acetyl-DL-methionine | 3.08 | 0.08 | < 0.001 | < 0.001 | 190.05 | 182.28 | Amino acids |
| Citrate | 1.02 | 6.43 | < 0.001 | < 0.001 | 191.02 | 509.34 | Tricarboxylic acid cycle metabolites |
| Adrenic Acid | 10.61 | 10.24 | < 0.001 | < 0.001 | 331.26 | 38.30 | Fatty Acids |
| D-Arabinono-1,4-lactone | 1.69 | 1.76 | < 0.001 | < 0.001 | 147.03 | 73.04 | Fatty Acids |
| (4Z,7Z,10Z,13Z,16Z,19Z)-4,7,10,13,1 6,19-Docosahexaenoic acid | 13.68 | 2.60 | < 0.001 | < 0.001 | 327.23 | 39.40 | Fatty Acids |
| 7Z, 10Z, 13Z, 16Z, 19Z-Docosapentaenoic acid | 8.73 | 2.98 | < 0.001 | < 0.001 | 329.25 | 38.43 | Fatty Acids |
| Oleic acid | 22.90 | 3.73 | < 0.001 | < 0.001 | 281.25 | 39.29 | Fatty Acids |
| 4-Pyridoxic acid | 1.33 | 0.13 | < 0.001 | < 0.001 | 182.05 | 39.81 | Pyridines and derivatives |
| Hydroxyphenyllactic acid | 1.10 | 3.50 | < 0.001 | < 0.001 | 181.05 | 177.85 | Carboxylic acids |
| N-Acetyl-L-aspartic acid | 1.27 | 0.35 | < 0.001 | < 0.001 | 174.04 | 419.22 | Amino acids |
| D-Ribose | 2.52 | 1.63 | < 0.001 | < 0.001 | 209.07 | 258.68 | Carbohydrates |
| Thymidine | 4.45 | 0.01 | < 0.001 | < 0.001 | 241.08 | 92.90 | Pyrimidine nucleosides |
| D-Fructose 1,6-bisphosphate | 2.20 | 0.05 | < 0.001 | < 0.001 | 399.01 | 456.50 | Carbohydrates |
| D-Allose | 1.86 | 2.56 | < 0.001 | < 0.001 | 359.12 | 276.82 | Carbohydrates |
| gamma-L-Glutamyl-L-phenylalanine | 1.59 | 0.05 | < 0.001 | < 0.001 | 293.11 | 365.03 | Dipeptides |
| 13-OxoODE | 1.86 | 0.39 | < 0.001 | < 0.001 | 293.21 | 42.64 | Fatty Acids |
| D-Galactarate | 1.89 | 1.56 | < 0.001 | < 0.001 | 191.02 | 71.61 | Carbohydrates |
| DL-3-Phenyllactic acid | 3.07 | 6.30 | < 0.001 | < 0.001 | 165.06 | 110.12 | Alkaloids |
| 16R-HETE | 4.66 | 3.22 | < 0.001 | < 0.001 | 319.23 | 43.72 | Fatty Acids |
| Stearic acid | 3.79 | 2.69 | < 0.001 | 0.001 | 283.26 | 39.55 | Fatty Acids |
| 2-Methylbenzoic acid | 1.03 | 1.58 | 0.001 | 0.001 | 135.04 | 99.78 | Carboxylic acids |
| Linoleic acid | 23.53 | 1.66 | 0.001 | 0.001 | 279.23 | 39.73 | Fatty Acids |
| Succinate | 1.95 | 0.19 | 0.002 | 0.002 | 117.02 | 376.74 | Tricarboxylic acid cycle metabolites |
| 2E-Eicosenoic acid | 3.34 | 4.71 | 0.002 | 0.003 | 309.28 | 38.47 | Fatty Acids |
| Palmitic acid | 6.94 | 1.96 | 0.002 | 0.003 | 253.22 | 40.36 | Fatty Acids |
| gamma-L-Glutamyl-L-valine | 1.48 | 0.06 | 0.004 | 0.004 | 245.11 | 399.87 | Amino acids |
| D-Maltose | 1.07 | 5.38 | 0.005 | 0.005 | 341.11 | 394.67 | Carbohydrates |
| 4-Hydroxy-3-methylbenzoic acid | 4.34 | 0.32 | 0.012 | 0.012 | 151.04 | 200.62 | Carboxylic acids |
| DL-lactate | 2.59 | 1.31 | 0.013 | 0.014 | 89.02 | 227.00 | Carboxylic acids |
| Erucic acid | 1.56 | 7.95 | 0.017 | 0.018 | 337.31 | 37.78 | Fatty Acids |
| Dodecanoic acid | 1.25 | 0.69 | 0.018 | 0.019 | 199.17 | 41.16 | Fatty Acids |
| 16-Hydroxypalmitic acid | 1.72 | 2.59 | 0.033 | 0.034 | 271.23 | 46.45 | Fatty Acids |
| Myristic acid | 4.19 | 1.51 | 0.046 | 0.047 | 227.20 | 40.71 | Fatty Acids |

^a^ Relative metabolite concentrations in PDAC tissue samples compared with noncancerous pancreatic tissue.

PE, Phosphoethanolamine; PC, phosphocholine.

**Table S4. Fifty-five identified differential metabolites between PDAC and Control (Ctr) using serum samples in training set**

| **Metabolite** | **VIP** | **Fold change**^a^ | ***p* value** | **FDR** | **Class** |
| --- | --- | --- | --- | --- | --- |
| Embelin | 1.59 | 2.19 | < 0.001 | < 0.001 | Benzoquinones |
| alpha-Tocopherol (Vitamin E) | 1.91 | 1.61 | < 0.001 | < 0.001 | Nutrients |
| Palmitic acid | 14.35 | 1.46 | < 0.001 | < 0.001 | Fatty Acids |
| Stearic acid | 2.52 | 1.95 | < 0.001 | < 0.001 | Fatty Acids |
| 16-Hydroxypalmitic acid | 1.50 | 1.54 | < 0.001 | < 0.001 | Fatty Acids |
| Succinate | 1.17 | 0.62 | < 0.001 | < 0.001 | Tricarboxylic acid cycle metabolites |
| Adrenic Acid | 1.50 | 1.36 | < 0.001 | < 0.001 | Fatty Acids |
| Heptadecanoic acid | 1.24 | 1.29 | < 0.001 | < 0.001 | Fatty Acids |
| Norethindrone Acetate | 1.43 | 1.85 | < 0.001 | < 0.001 | Norethindrone |
| Confertifoline | 3.40 | 0.90 | < 0.001 | < 0.001 | Hydrocarbons |
| Linoleic acid | 13.83 | 1.30 | < 0.001 | < 0.001 | Fatty Acids |
| 2'-Deoxy-D-ribose | 3.23 | 1.48 | < 0.001 | 0.001 | Carbohydrates |
| Taurine | 1.86 | 0.83 | < 0.001 | 0.001 | Amino acids |
| D-Proline | 1.42 | 0.73 | < 0.001 | 0.001 | Amino acids |
| 2-Oxoadipic acid | 2.24 | 0.70 | 0.001 | 0.001 | Keto acids and derivatives |
| Myristic acid | 2.24 | 1.26 | 0.001 | 0.002 | Fatty Acids |
| L-Norleucine | 3.57 | 0.76 | 0.001 | 0.002 | Amino acids |
| DL-lactate | 1.98 | 0.80 | 0.001 | 0.002 | Hydroxy acids and derivatives |
| D(-)-beta-hydroxy butyric acid | 2.55 | 0.49 | 0.002 | 0.004 | Amino acids |
| 1-Palmitoyl-2-hydroxy-sn-glycero-3-PE | 1.38 | 1.31 | 0.003 | 0.005 | Phospholipids |
| 2-hydroxy-butanoic acid | 1.26 | 1.29 | 0.004 | 0.006 | Amino acids |
| alpha-Linolenic acid | 3.17 | 1.29 | 0.004 | 0.007 | Fatty Acids |
| Arachidonic Acid (peroxide free) | 3.37 | 0.86 | 0.004 | 0.007 | Fatty Acids |
| 2-Hydroxy-3-methylbutyric acid | 1.41 | 0.49 | 0.007 | 0.011 | Fatty Acids |
| Cholesterol 3-sulfate | 8.80 | 1.75 | 0.007 | 0.011 | Lipids |
| Taurochenodeoxycholate | 5.15 | 0.09 | 0.009 | 0.013 | Lipids |
| L-Glutamate | 2.55 | 0.55 | 0.009 | 0.013 | Amino acids |
| D-Fructose | 4.99 | 0.77 | 0.009 | 0.013 | Glucose |
| Indoxyl sulfate | 5.57 | 1.45 | 0.011 | 0.015 | Heterocyclic Compounds |
| Glycocholic acid | 10.16 | 0.33 | 0.011 | 0.015 | Lipids |
| Glycochenodeoxycholate | 10.34 | 0.35 | 0.012 | 0.015 | Lipids |
| cis-9-Palmitoleic acid | 3.91 | 1.27 | 0.015 | 0.019 | Fatty Acids |
| L-Phenylalanine | 2.60 | 0.83 | 0.018 | 0.021 | Amino acids |
| Hippuric acid | 1.28 | 1.91 | 0.025 | 0.028 | Keto Acids |
| Oleic acid | 3.46 | 1.68 | 0.028 | 0.031 | Fatty Acids |
| L-Tryptophan | 1.60 | 0.84 | 0.029 | 0.031 | Amino acids |
| ketoisocaproic acid | 3.13 | 0.79 | 0.030 | 0.032 | Keto acids and derivatives |
| m-Chlorohippuric acid | 2.01 | 0.81 | 0.049 | 0.049 | Keto Acids |
| Alpha-D-Glucose | 5.53 | 0.89 | 0.049 | 0.049 | Glucose |
| epsilon-Caprolactam | 1.11 | 1.39 | < 0.001 | < 0.001 | Amides |
| 2-Ethoxyethanol | 1.88 | 1.61 | < 0.001 | < 0.001 | Alcohols |
| Sphingomyelin (d18:1/18:0) | 1.85 | 0.79 | < 0.001 | < 0.001 | Lipids |
| L-Glutamine | 1.91 | 1.40 | < 0.001 | < 0.001 | Amino acids |
| L-Carnitine | 2.72 | 1.41 | < 0.001 | < 0.001 | Lipids |
| Thioetheramide-PC | 2.37 | 0.56 | < 0.001 | < 0.001 | Phospholipids |
| L-Pyroglutamic acid | 1.45 | 1.26 | < 0.001 | < 0.001 | Amino acids |
| L-Isoleucine | 1.53 | 0.79 | < 0.001 | 0.0002 | Amino acids |
| 1-Stearoyl-sn-glycerol | 1.01 | 0.92 | < 0.001 | 0.001 | Lipids |
| PC(16:0/16:0) | 4.13 | 0.55 | 0.001 | 0.002 | Phospholipids |
| 1,2-dioleoyl-sn-glycero-3-phosphatidylcholine | 3.73 | 0.69 | 0.002 | 0.003 | Phospholipids |
| 1-Stearoyl-sn-glycerol 3-PC | 2.13 | 1.22 | 0.006 | 0.009 | Phospholipids |
| Creatine | 1.41 | 0.69 | 0.016 | 0.019 | Amino acids |
| L-Pipecolic acid | 1.08 | 1.26 | 0.017 | 0.020 | Carboxylic acids and derivatives |
| 1-Palmitoylglycerol | 1.28 | 0.95 | 0.027 | 0.031 | Lipids |
| Hypoxanthine | 1.20 | 1.14 | 0.042 | 0.044 | Alkaloids |

^a^ Relative concentrations in PDAC serum samples compared with controls.

PE, Phosphoethanolamine; PC, phosphocholine.

**Table S5. Perturbed metabolic pathways commonly found in PDAC tissue and serum metabolomics analysis in the comparison of PDAC and NT or Ctr**

|  | PDAC vs NT (Tissue) | | | PDAC vs Ctr (Serum) | | |
| --- | --- | --- | --- | --- | --- | --- |
| Map.Name | Test | *P* value | Rich Factor | Test | *P* value | Rich Factor |
| Central carbon metabolism in cancer | 22 | < 0.001 | 0.595 | 7 | < 0.001 | 0.1892 |
| Protein digestion and absorption | 35 | < 0.001 | 0.273 | 6 | < 0.001 | 0.1277 |
| Mineral absorption | 23 | < 0.001 | 0.489 | 5 | < 0.001 | 0.1724 |
| Aminoacyl-tRNA biosynthesis | 20 | < 0.001 | 0.385 | 6 | < 0.001 | 0.1154 |
| GABAergic synapse | 14 | < 0.001 | 0.483 | 3 | < 0.001 | 0.189 |
| Biosynthesis of unsaturated fatty acids | 10 | < 0.001 | 0.357 | 6 | < 0.001 | 0.128 |
| Fatty acid biosynthesis | 6 | < 0.001 | 0.261 | 5 | 0.001 | 0.172 |
| Alanine, aspartate and glutamate metabolism | 4 | < 0.001 | 0.444 | 3 | 0.004 | 0.115 |
| ABC transporters | 6 | 0.014 | 0.120 | 6 | 0.004 | 0.333 |
| Arginine biosynthesis | 6 | 0.020 | 0.111 | 2 | 0.028 | 0.081 |

**Table S6. Fourteen overlapping differential metabolites in tissue and serum metabolomics analysis (PDAC vs NT in tissue and PDAC vs Ctr and PDAC vs BP in serum)**

| Metabolite | VIP | |  | p-value | |  | AUC | |  | Fold change | |
| --- | --- | --- | --- | --- | --- | --- | --- | --- | --- | --- | --- |
|  | tissue | serum |  | tissue | serum |  | tissue | serum |  | tissue | serum |
| Taurine | 10.060 | 1.861 |  | < 0.001 | < 0.001 |  | 0.959 | 0.684 |  | 2.836🡑 | 0.828🡓 |
| L-Tryptophan | 7.112 | 1.602 |  | < 0.001 | 0.029 |  | 0.947 | 0.611 |  | 0.250🡓 | 0.838🡓 |
| L-Glutamate | 1.723 | 1.276 |  | < 0.001 | 0.027 |  | 0.901 | 0.624 |  | 0.393🡓 | 0.715🡓 |
| L-Glutamine | 1.292 | 1.914 |  | < 0.001 | < 0.001 |  | 0.923 | 0.802 |  | 0.261🡓 | 1.402🡑 |
| D-Proline | 2.863 | 1.423 |  | < 0.001 | < 0.001 |  | 0.943 | 0.698 |  | 0.260🡓 | 0.727🡓 |
| Arachidonic Acid | 2.314 | 3.368 |  | < 0.001 | 0.004 |  | 0.834 | 0.635 |  | 1.995🡑 | 0.859🡓 |
| L-Pyroglutamic acid | 1.360 | 1.453 |  | 0.001 | < 0.001 |  | 0.925 | 0.793 |  | 0.043🡓 | 1.259🡑 |
| Creatine | 1.868 | 1.408 |  | 0.041 | 0.016 |  | 0.540 | 0.668 |  | 0.712🡓 | 0.695🡓 |
| L-Isoleucine | 1.822 | 1.532 |  | < 0.001 | < 0.001 |  | 0.962 | 0.730 |  | 0.172🡓 | 0.793🡓 |
| Adrenal Acid | 10.608 | 1.501 |  | < 0.001 | < 0.001 |  | 0.950 | 0.729 |  | 10.240🡑 | 1.358🡑 |
| Linoleic acid | 23.529 | 13.83 |  | 0.001 | < 0.001 |  | 0.749 | 0.696 |  | 1.658🡑 | 1.305🡑 |
| Palmitic acid | 6.940 | 14.353 |  | 0.002 | < 0.001 |  | 0.727 | 0.8 |  | 1.955🡑 | 1.465🡑 |
| 16-Hydroxypalmitic acid | 1.722 | 1.502 |  | 0.033 | < 0.001 |  | 0.774 | 0.772 |  | 2.588🡑 | 1.544🡑 |
| Myristic acid | 4.185 | 2.238 |  | 0.046 | 0.001 |  | 0.625 | 0.728 |  | 1.508🡑 | 1.258🡑 |

*Fold change*, relative concentrations in PDAC serum samples compared with control in tissue or serum samples.

🡓 means down-regulated; 🡑 means up-regulated.

**Table S7. ANOVA results using the data of the five potential biomarker metabolites in serum metabolomics analysis**

| Metabolite | PDAC vs Ctr (ANOVA) | | | | | |
| --- | --- | --- | --- | --- | --- | --- |
|  | F value | P value | Gender | age | diabetes | hypertension |
| L-Tryptophan | 12.408 | < 0.001 | 0.738 | 0.380 | 0.816 | 0.719 |
| D-Proline | 18.486 | < 0.001 | 0.155 | 0.581 | 0.732 | 0.832 |
| Creatine | 5.782 | 0.018 | 0.114 | 0.287 | 0.208 | 0.839 |
| Arachidonic acid | 12.408 | < 0.001 | 0.738 | 0.380 | 0.816 | 0.719 |
| Palmitic acid | 35.849 | < 0.001 | 0.995 | 0.099 | 0.860 | 0.436 |

**Table S8. Logistic regression analysis reveals the biomarker panel was independent of the possible confounding factors including age, gender and history of diabetes.**

|  | Coefficient | Standard error | *p*-value |
| --- | --- | --- | --- |
| Intercept | -1.748 | 2.075 | 0.400 |
| Biomarker | 5.991 | 1.194 | < 0.001 |
| Gender | 0.738 | 0.517 | 0.153 |
| Age | -0.035 | 0.035 | 0.315 |
| Diabetes | 13.857 | 175.007 | 0.937 |
| Hypertension | 2.234 | 0.846 | 0.008 |

**Table S9. Performance of the biomarker model in the training set and validation set in the serum metabolomics analysis**

|  | **Training set** | | | | |  | | **Validation set** | | | |  |
| --- | --- | --- | --- | --- | --- | --- | --- | --- | --- | --- | --- | --- |
|  | AUC (95%CI) | Sensitivity | | Specificity | Accuracy | |  | AUC (95%CI) | Sensitivity | Specificity | Accuracy | |
| **PDAC vs Ctr** |  | |  |  |  |  | |  |  |  |  |  |
| Biomarker | 0.854 (0.842-0.865) | | 0.800 | 0.792 | 0.797 |  | |  |  |  |  |  |
| Biomarker & CA19-9 | 0.919 (0.911-0.928) | | 0.825 | 0.896 | 0.852 |  | |  |  |  |  |  |
| **Early-stage PDAC vs Ctr** |  | |  |  |  |  | |  |  |  |  |  |
| Biomarker | 0.880 (0.864-0.896) | | 0.889 | 0.792 | 0.833 |  | | 0.830 (0.793-0.866) | 0.762 | 0.704 | 0.729 |  |
| Biomarker & CA19-9 | 0.900 (0.886-0.915) | | 0.861 | 0.854 | 0.857 |  | | 0.949 (0.933-0.966) | 0.857 | 0.815 | 0.833 |  |
| **PDAC vs BP** |  | |  |  |  |  | |  |  |  |  |  |
| CA19-9 | 0.806 (0.719-0.892) | | 0.750 | 0.861 | 0.722 |  | | 0.757 (0.616-0.897) | 0.773 | 0.741 | 0.735 |  |
| Biomarker | 0.865 (0.800-0.931) | | 0.763 | 0.861 |  |  | | 0.852 (0.736-0.967) | 0.864 | 0.778 |  |  |
| Biomarker & CA19-9 | 0.917 (0.868-0.966) | | 0.863 | 0.861 |  |  | | 0.909 (0.825-0.993) | 0.818 | 0.889 |  |  |
